# Supplementary material for: Global genomic analyses of wheat powdery mildew reveal association of pathogen spread with historical human migration and trade
Source: Nat Commun. 2022 Jul 26;13:4315. doi: 10.1038/s41467-022-31975-0 (PMC9315327; doi:10.1038/s41467-022-31975-0)
Supplement: Supplementary file 1 — Supplementary_Information [file 41467_2022_31975_MOESM1_ESM.pdf]

Supplementary Information

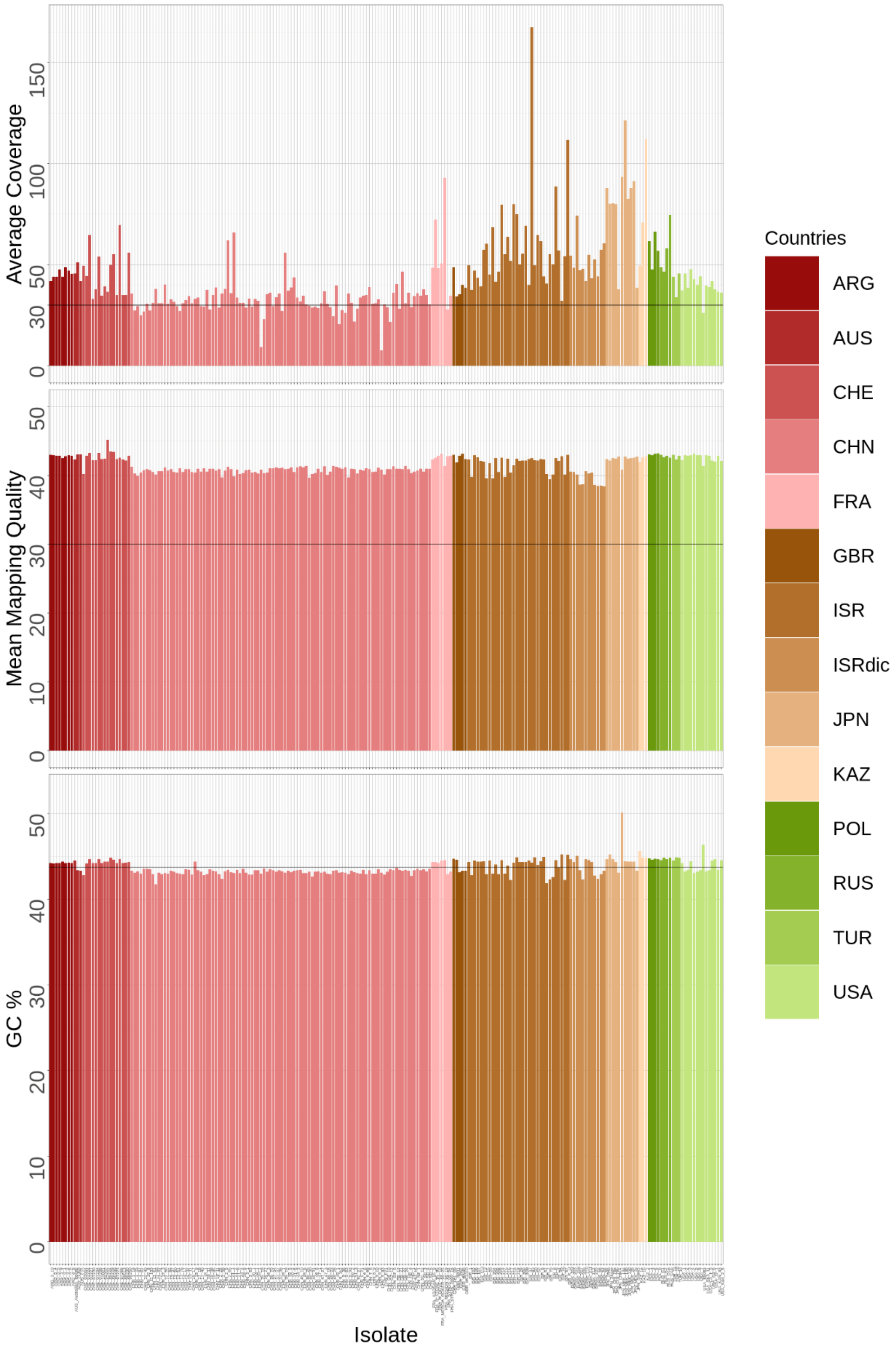

Supplementary Fig. 1. Analysis of the sequencing quality of isolates. (a) Average coverage of each bam file. **(b)** Mean mapping quality with a threshold of 30. **(c)** GC% percentage of all individuals across the genome from bam files using Qualimap with the threshold line of the average around 43. Isolate JPN_MEK_13a has an outlier GC% value, possibly due to a sequencing artifact. Source data are provided as a Source Data file.

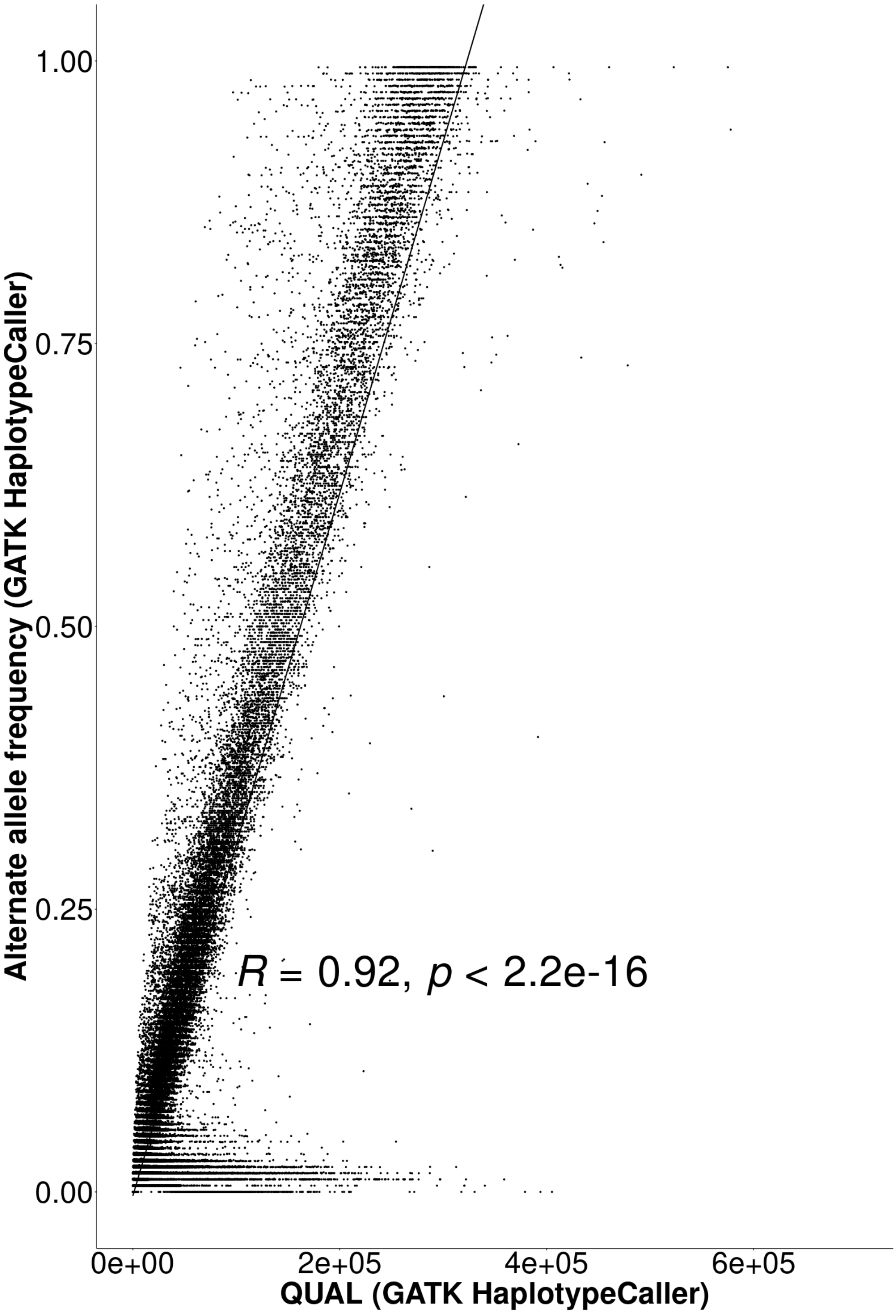

Supplementary Fig. 2. Correlation of the Alternate Allele Frequency with the QUAL (quality) of the SNPs across the entire genome (n=1,865,441), using the dataset with the *B. graminis* outgroups to define the ancestral alleles. The value of the Pearson's correlation test (two-sided, with no adjustments made for multiple comparisons) is 0.92 with a low p-value < 2.2e-16, indicating a strong positive correlation. Source data are provided as a Source Data file.

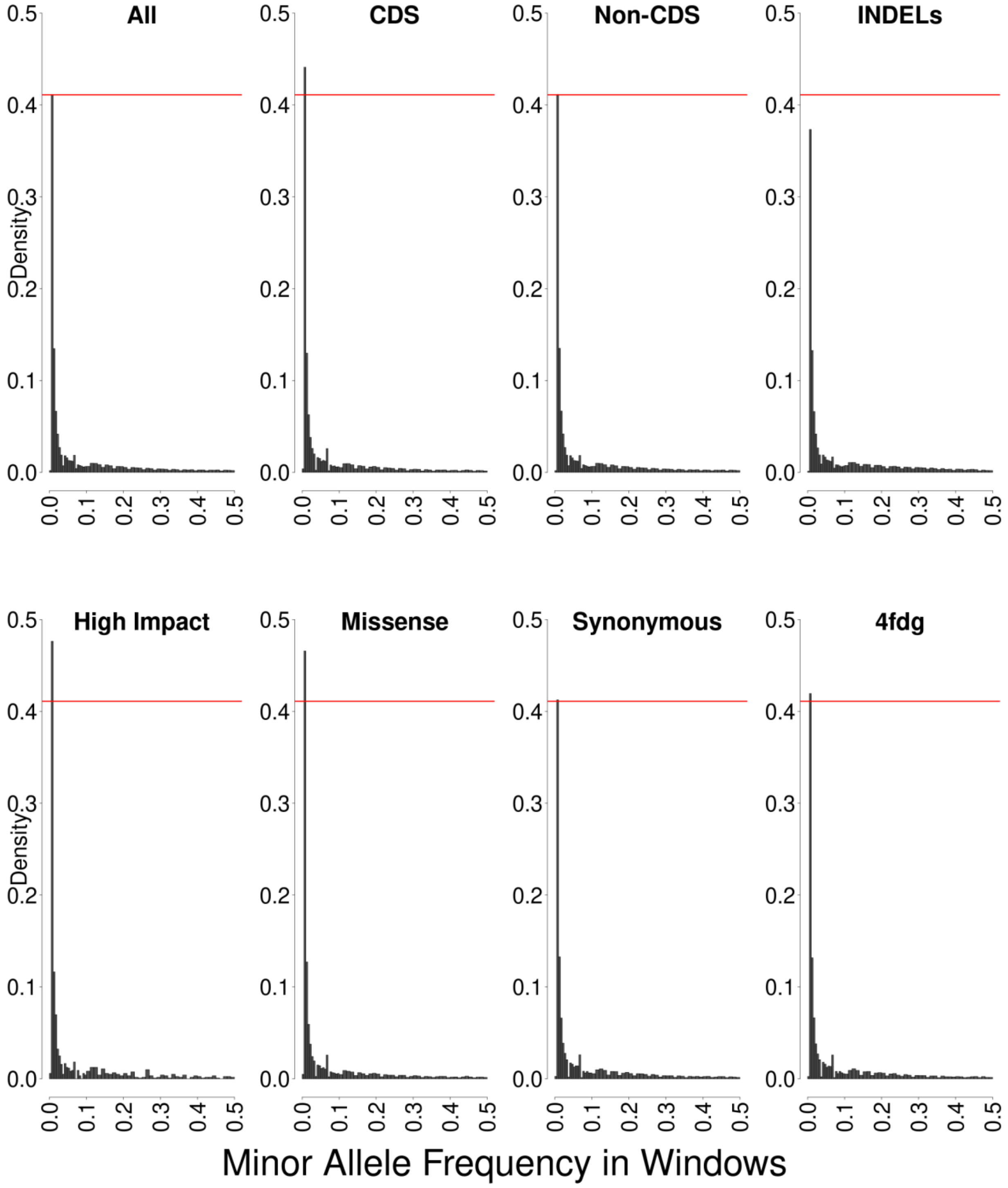

Supplementary Fig. 3. Density of allele frequencies for various subsites using the dataset with 172 isolates, filtered and with 90% maximum missing per site. All of them are on SNPs, except from the INDELs one. The red line has the height of the value of the first window in the Synonymous SNPs group. High impact, missense mutations and SNPs in the coding sequence are above the line for the lowest frequencies, implying that there the SNPs tend to be less widespread. Source data are provided as a Source Data file.

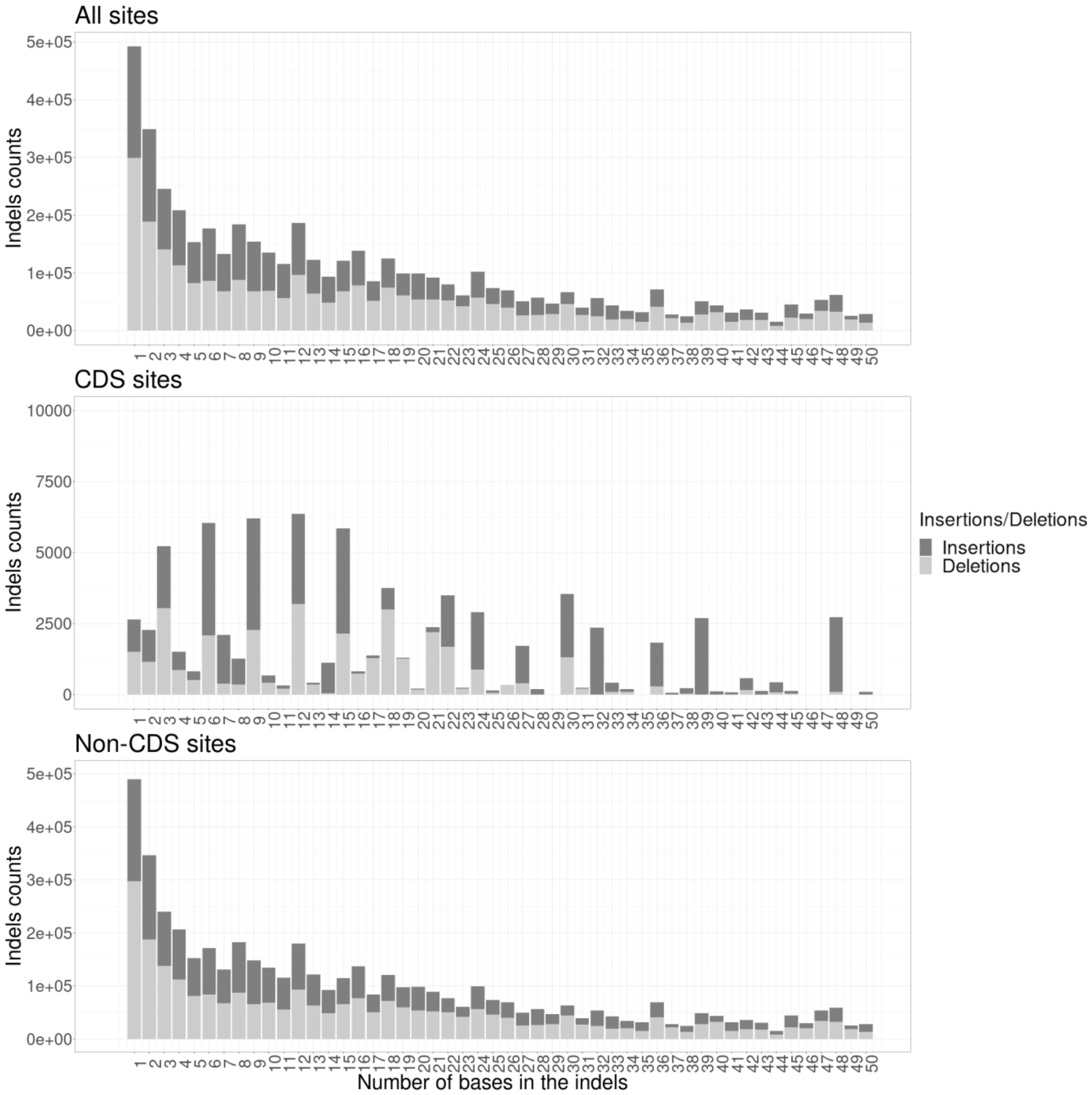

Supplementary Fig. 4. Length of INDELs in all sites, in CDS sites and non-CDS sites using the dataset with 172 isolates, filtered and with 90% maximum missing per site. The dark gray color is indicating insertions and the light gray deletions. Source data are provided as a Source Data file.

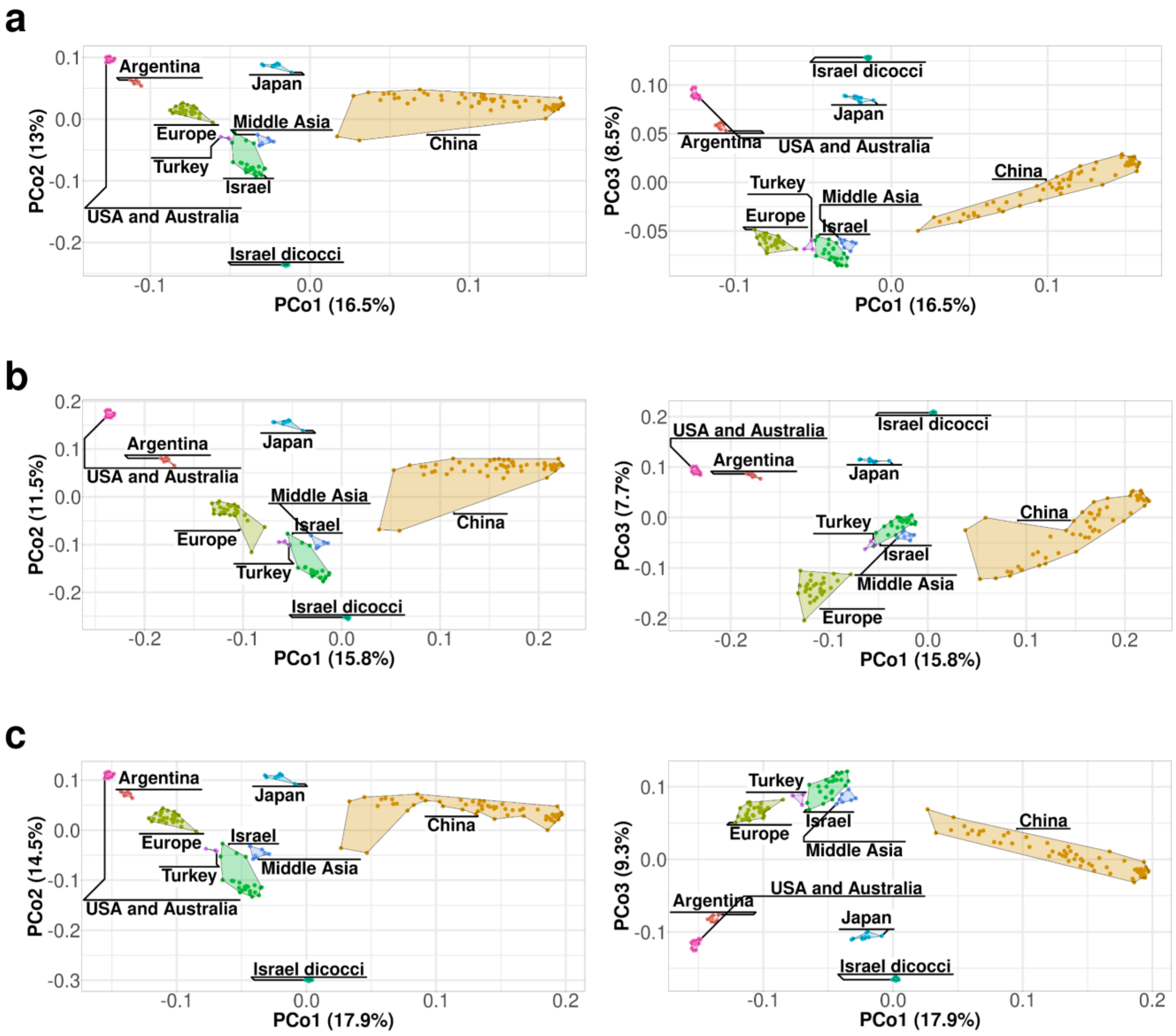

Supplementary Fig. 5. Combinations of the first three components of the PCoA analysis for the SNPs dataset of the 172 isolates after being filtered for their quality, minor allele frequency and LD pruned. PCoA was performed with regard to the **(a)** simple mismatch, **(b)** Bray-Curtis, and **(c)** Nei's genetic distances.

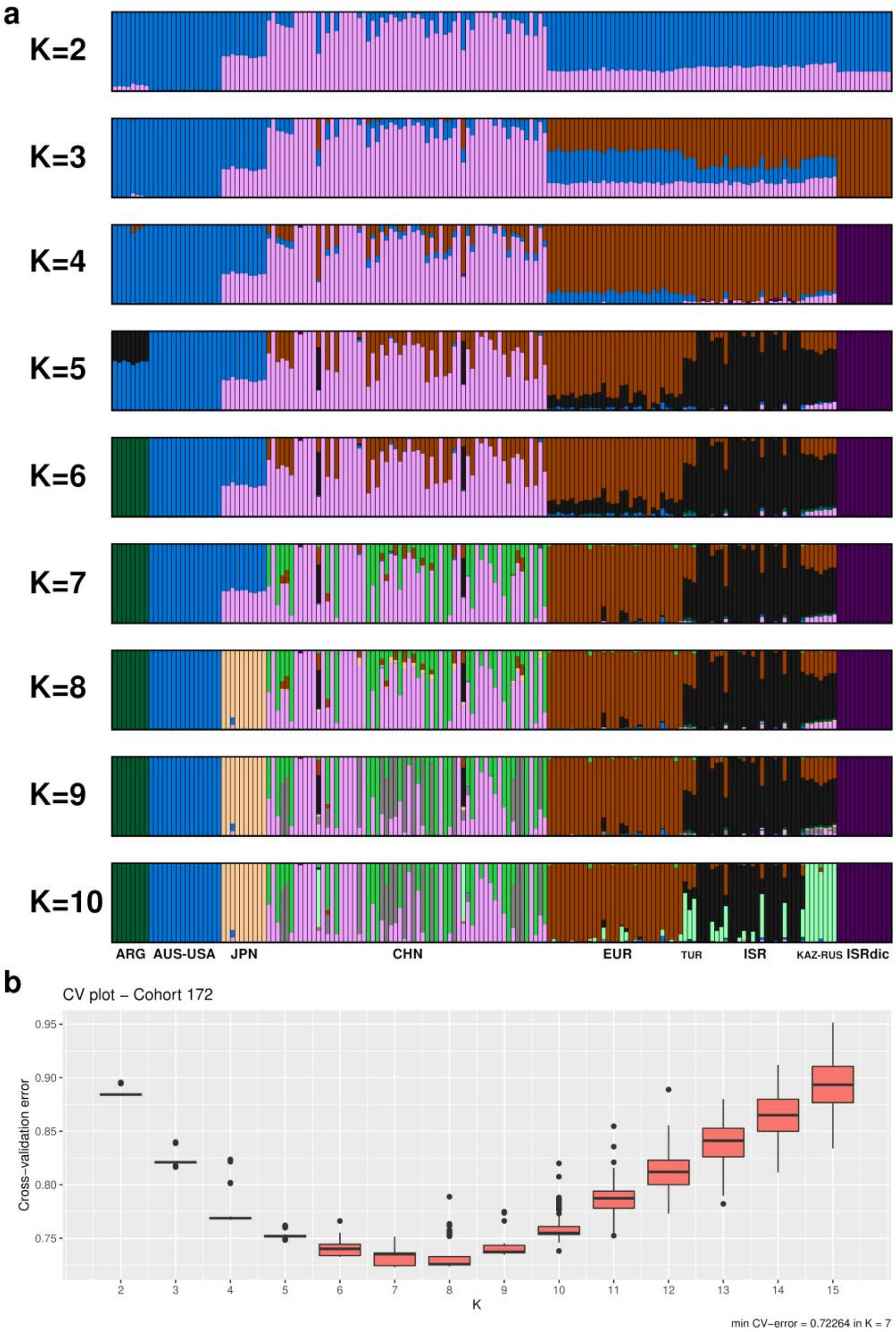

Supplementary Fig. 6. Admixture analysis and cross validation error statistics, using the Admixture software, 172 isolates, using LDpruned SNPs and strictly excluding clonal and unqualified SNPs and isolates. We analysed for K=2 up to K=10. TUR, ISR, KAZ and RUS mildew isolates together are characterized as Mid-Asian and all the isolates from populations in countries from Europe and Asia are termed Eurasian. **(a)**The results of the most common ancestral patterns from K=2 to K=10, using the cohort with 172 isolates, 100 repetitions and drawn with the software Pong (n=100). **(b)** Cross-Validation error plot for K=2 to K=15, showing that the most supported Ks are no more than K=8. Boxes indicate the inter-quartile range (IQR) with the central line indicating the median and whiskers indicating the minimum and maximum without outliers, respectively. Outliers were defined as minimum – 1.5*IQR and maximum + 1.5*IQR, respectively. Source data are provided as a Source Data file.

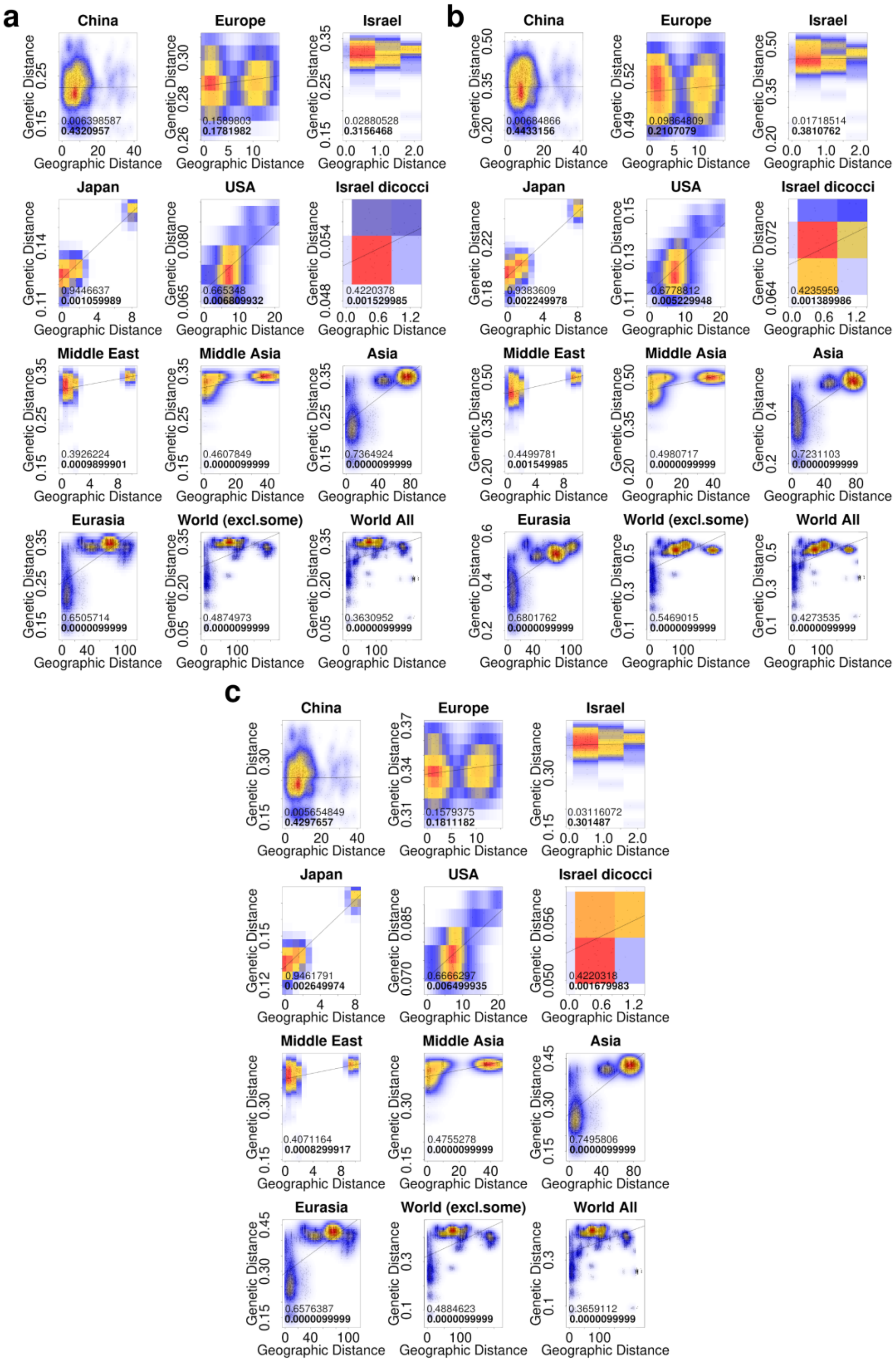

Supplementary Fig. 7. Association of the Geographic Distance (tens of kilometers by coordinates of the isolates) with various genetic distances using Kernel Density Estimation. In each plot there is also depicted the Mantel test correlation and its p-value in bold below that. The results are shown for the **(a)** simple mismatch, **(b)** Bray-Curtis, and **(c)** Nei's genetic distances.

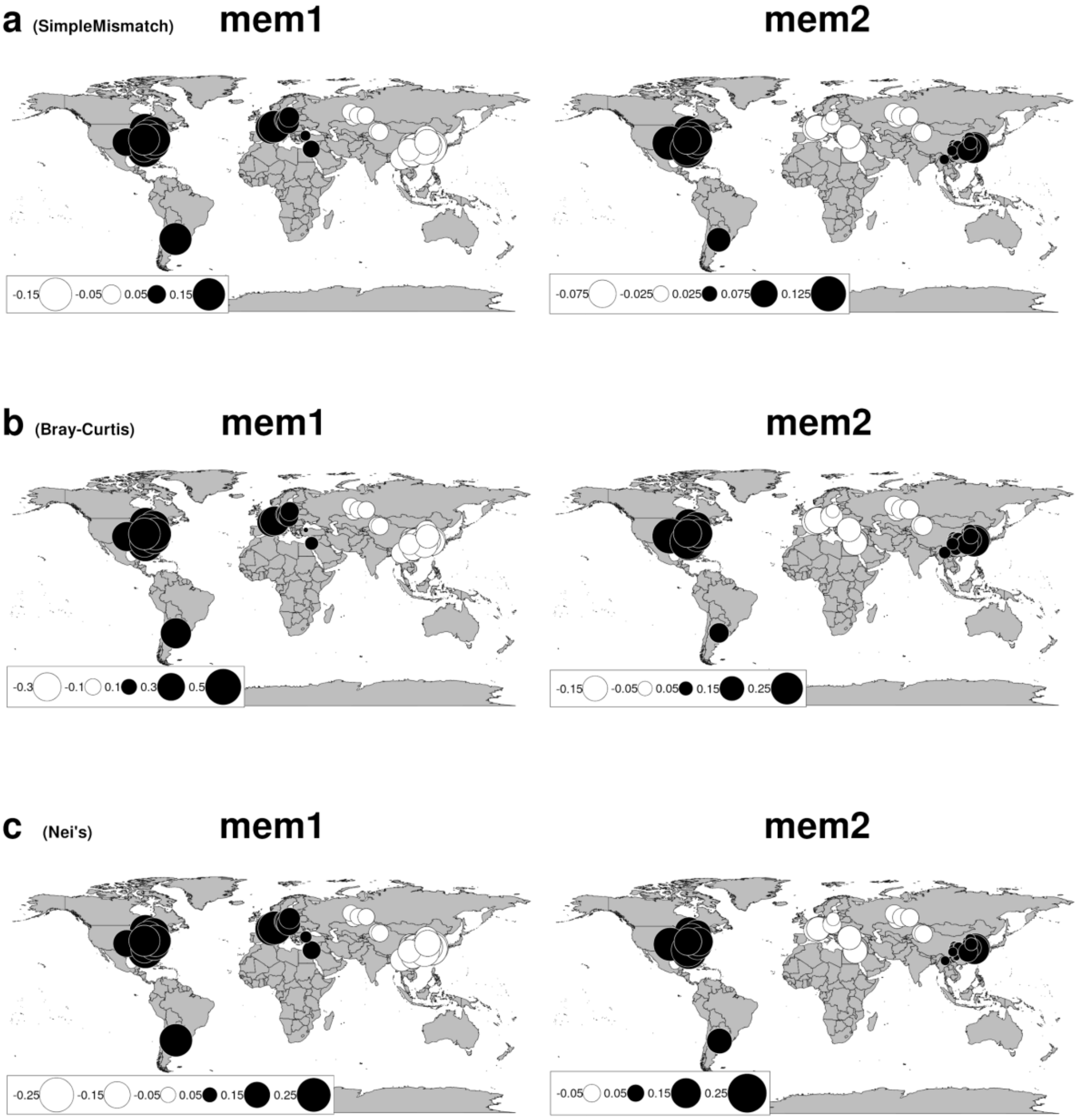

Supplementary Fig. 8. Analysis of factorial dbMEM using the worldwide dataset of 172 isolates (excluding isolates from Australia and Japan since they are either transferred there quite recently or they are recent recombinants) where there were known coordinates. This method is used to illustrate spatial correlation and/or separation regarding one eigenvector (e.g. geographical barriers). The two levels of dbMEM eigenfunctions with the highest impact are presented. The black and white shades and its various sizes indicate the difference between the different points (isolates) in relation to their position on the map. The larger the dot the bigger the positive (black) or negative (white) spatial correlation between isolates for each dbMEM eigenfunction. The first eigenvector (mem1) separates East and Middle Asian mildew isolates from the rest. The second eigenvector (mem2) separates American mostly (and Chinese) mildew isolates from the rest of the Eurasian isolates. The results are shown for the **(a)** simple mismatch, **(b)** Bray-Curtis, and **(c)** Nei's genetic distances.

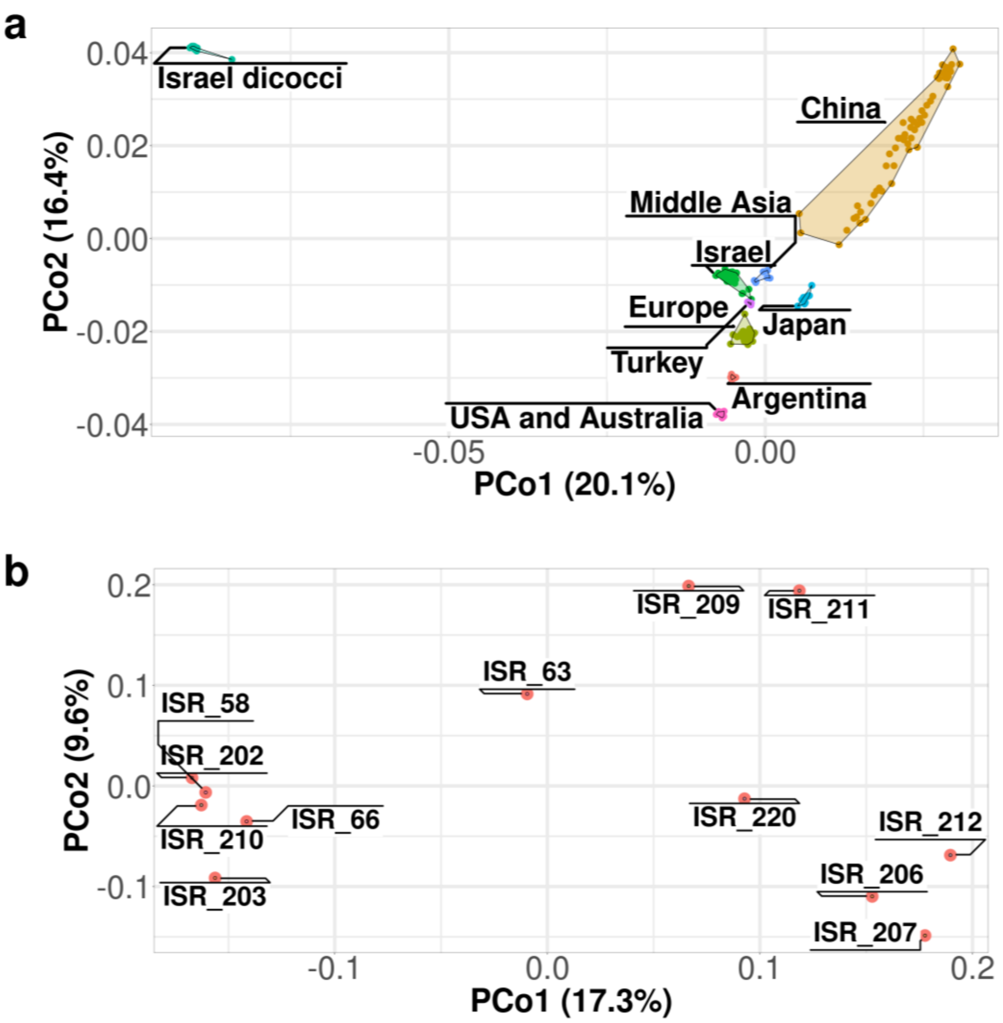

Supplementary Fig. 9. PCoA analysis for the SNPs dataset of the 172 isolates after being filtered for their quality and minor allele frequency, using simple mismatch distances. **(a)** PCoA of the cohort with all 172 *B.g. tritici* and *B.g. dicocci* isolates using non LD-pruned SNPs. *B.g. dicocci* isolates separate clearly from all *B.g. tritici* isolates. Notably, *B.g. dicocci* isolates separate into subgroups which are not visible when LD pruned SNPs are used (see Figure 1b). **(b)** PCoA using only *B.g. dicocci* isolates and LD-pruned SNPs. The *B.g. dicocci* isolates split into distinct groups, suggesting the presence of multiple distinct genetic lineages.

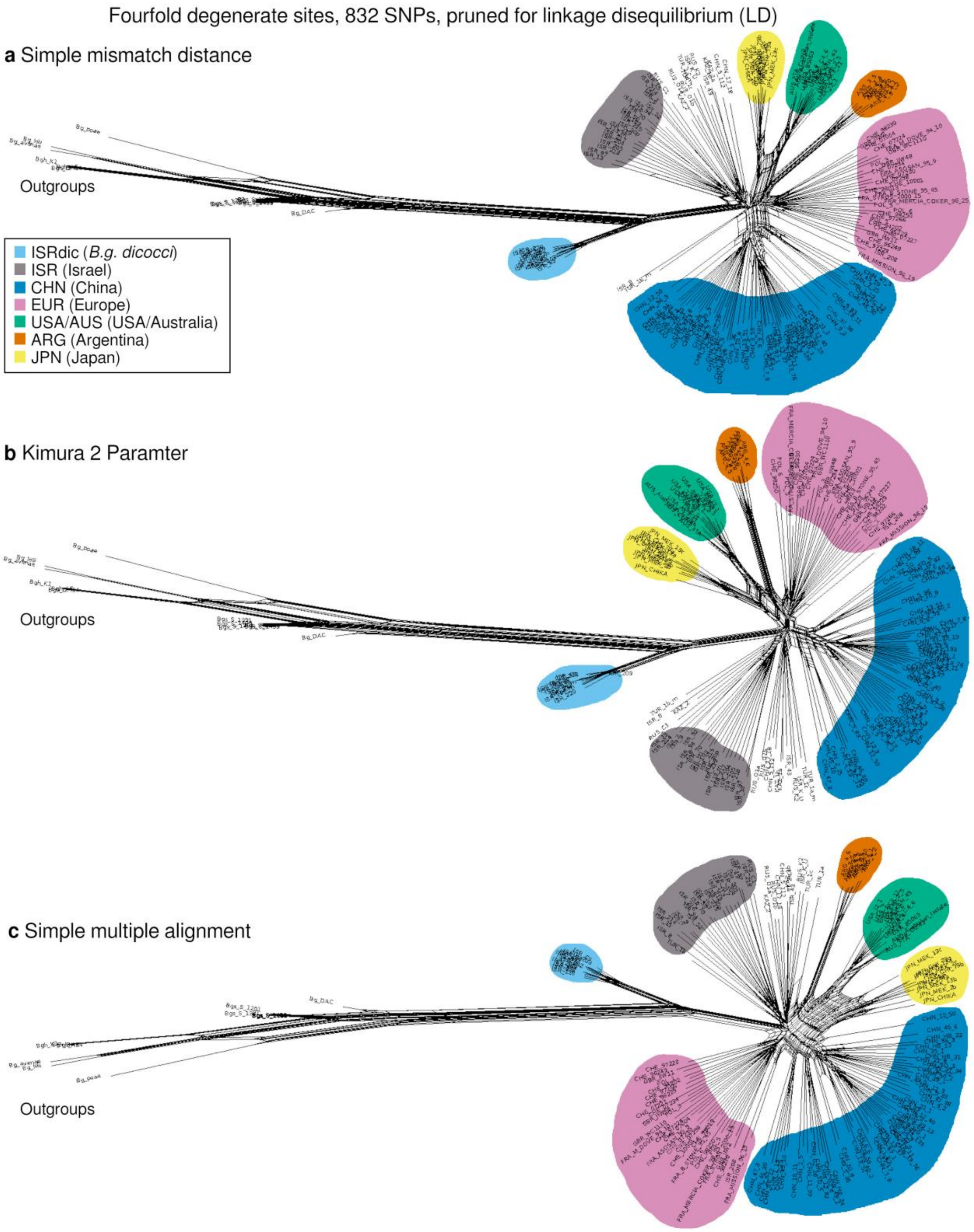

Supplementary Fig. 10. Phylogenetic Networks using NeighborNet in Splitstree using the cohort with 184 isolates (including the outgroups) and only 4fdg sites, biallelic and LD-pruned SNPs, excluding indels and SNPs in chromosome Bgt_chr-Un. Missing data SNPs were also excluded. To construct the networks we used: **(a)** Simple mismatch individual distances **(b)** the Kimura 2-parameter model to get distances from SNPs and **(c)** a single multiple alignment of SNPs. Some of the non-coloured individuals are mildew isolates from Russia, Kazakhstan, Turkey, Israel and China.

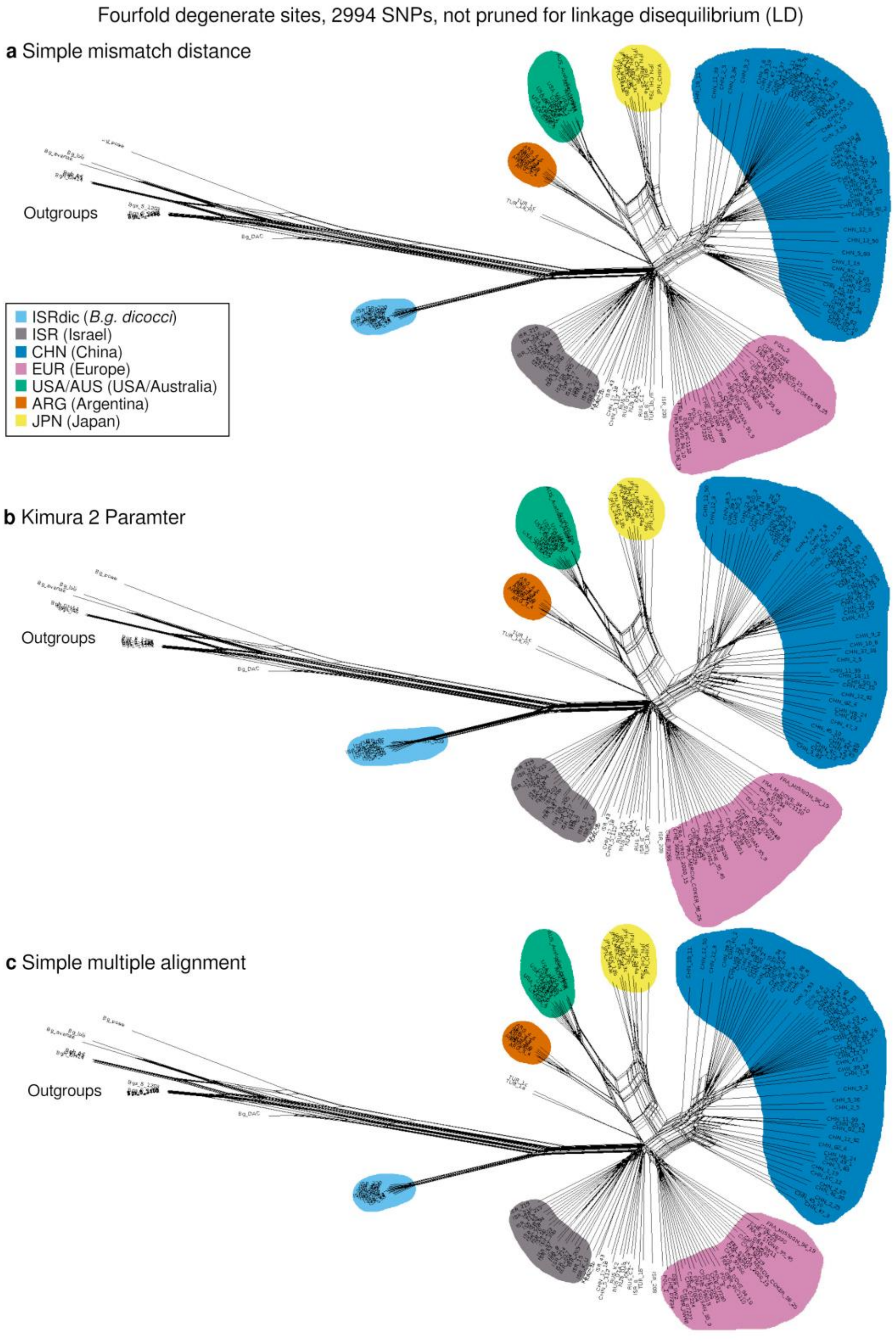

Supplementary Fig. 11. Phylogenetic Networks using NeighborNet in Splitstree using the cohort with 184 isolates (including the outgroups) and only 4fdg sites, biallelic and non LD-pruned SNPs, excluding indels and SNPs in chromosome Bgt_chr-Un. Missing data SNPs were also excluded. To construct the networks we used: **(a)** Simple mismatch individual distances **(b)** the Kimura 2-parameter model to get distances from SNPs and **(c)** a single multiple alignment of SNPs. Some of the non-coloured individuals are mildew isolates from Russia, Kazakhstan, Turkey, Israel and China.

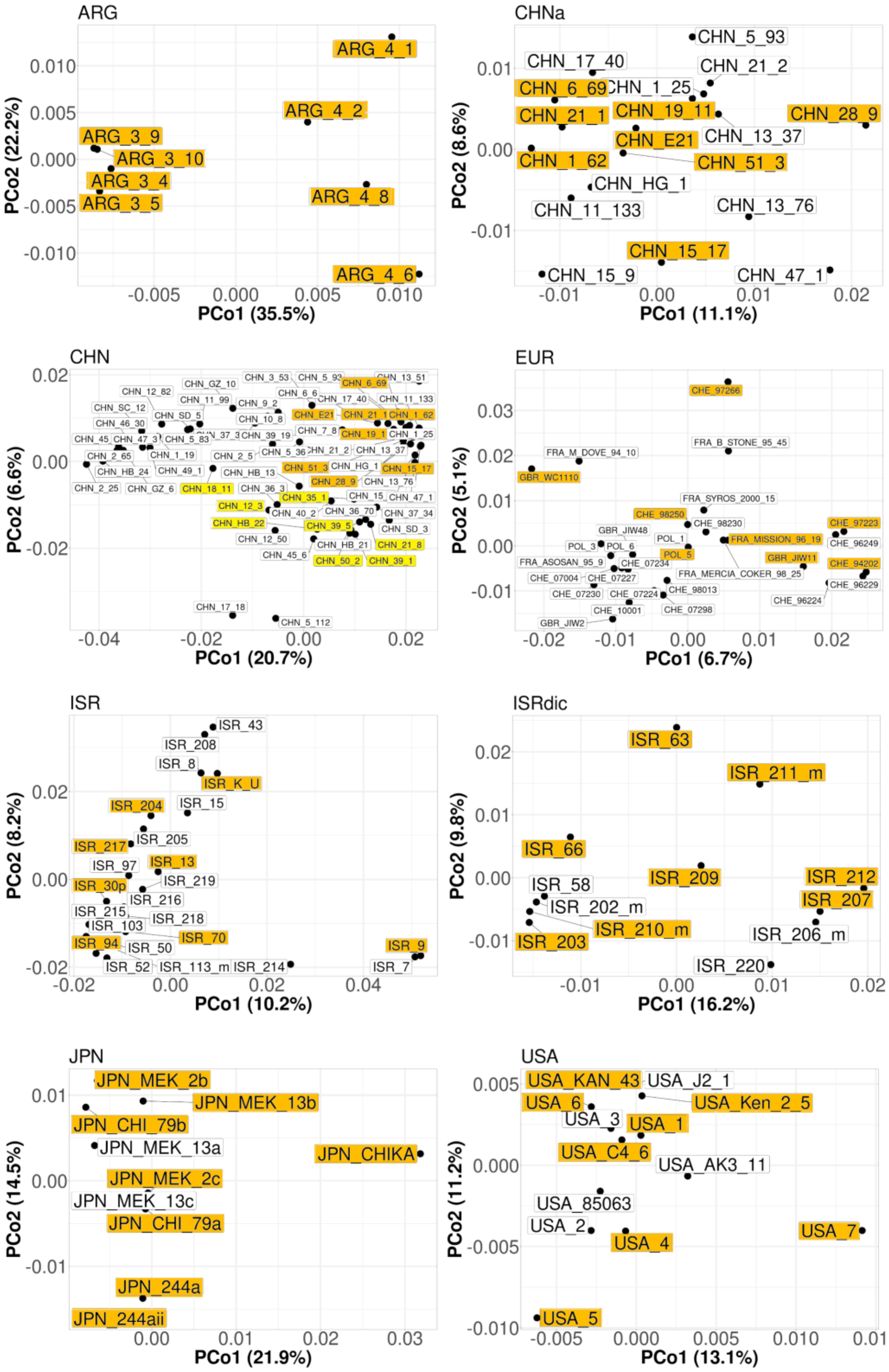

Supplementary Fig. 12. PCoA using LD pruned SNP data for each population. We picked eight isolates with big Simple Mismatch distances between them per population (ARG, CHNa, CHN, EUR, ISR, ISRdic, JPN and USA) for analyses such as the coalescence. The selected isolates are highlighted in orange. For Chinese isolates, two PCoAs are shown, one with only the putative ancestral isolates (CHNa), and one with all isolates (CHN) in which the putative hybrids (CHNh) are highlighted in yellow and CHNa in orange.

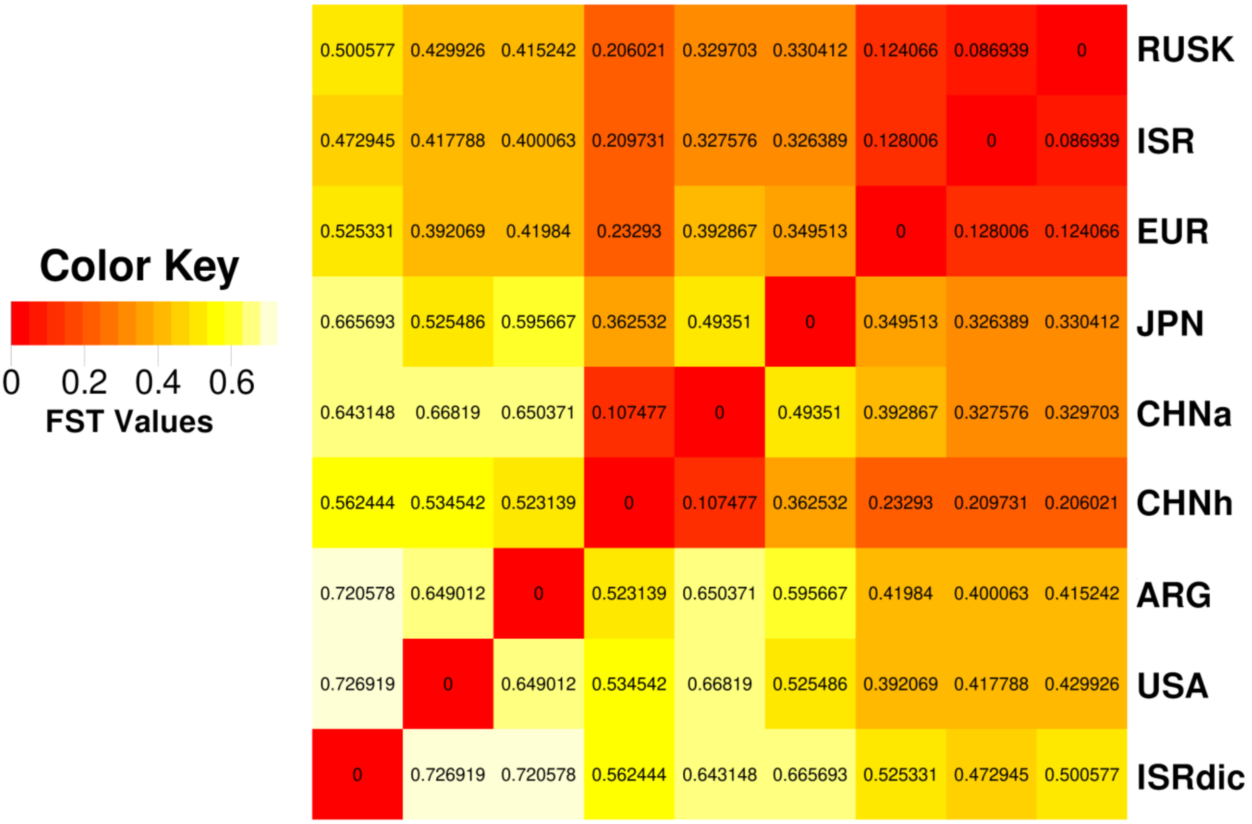

Supplementary Fig. 13. Fixation Index between all populations (average over the whole genome), using PopGenome in R. A value of zero implies complete panmixis, where the populations can interbreed freely, while a value of one implies that the population structure can explain all the genetic variation between the populations and that they do not share any genetic diversity. The data is displayed as a heat map for better visibility of differences, with the numeric values in each respective square. The Eurasian *B.g. tritici* populations are closer to zero, indicating a state close to panmixis, while the ARG, USA and *B.g. dicocci* populations seem to be more isolated with F_{ST} values of roughly 0.4-0.7. Chinese mildew populations are either with all isolates (CHN), only the ones with the East Asian ancestry (CHNa) or only the ones with at least 25% European ancestry from ADMIXTURE (CHNh).

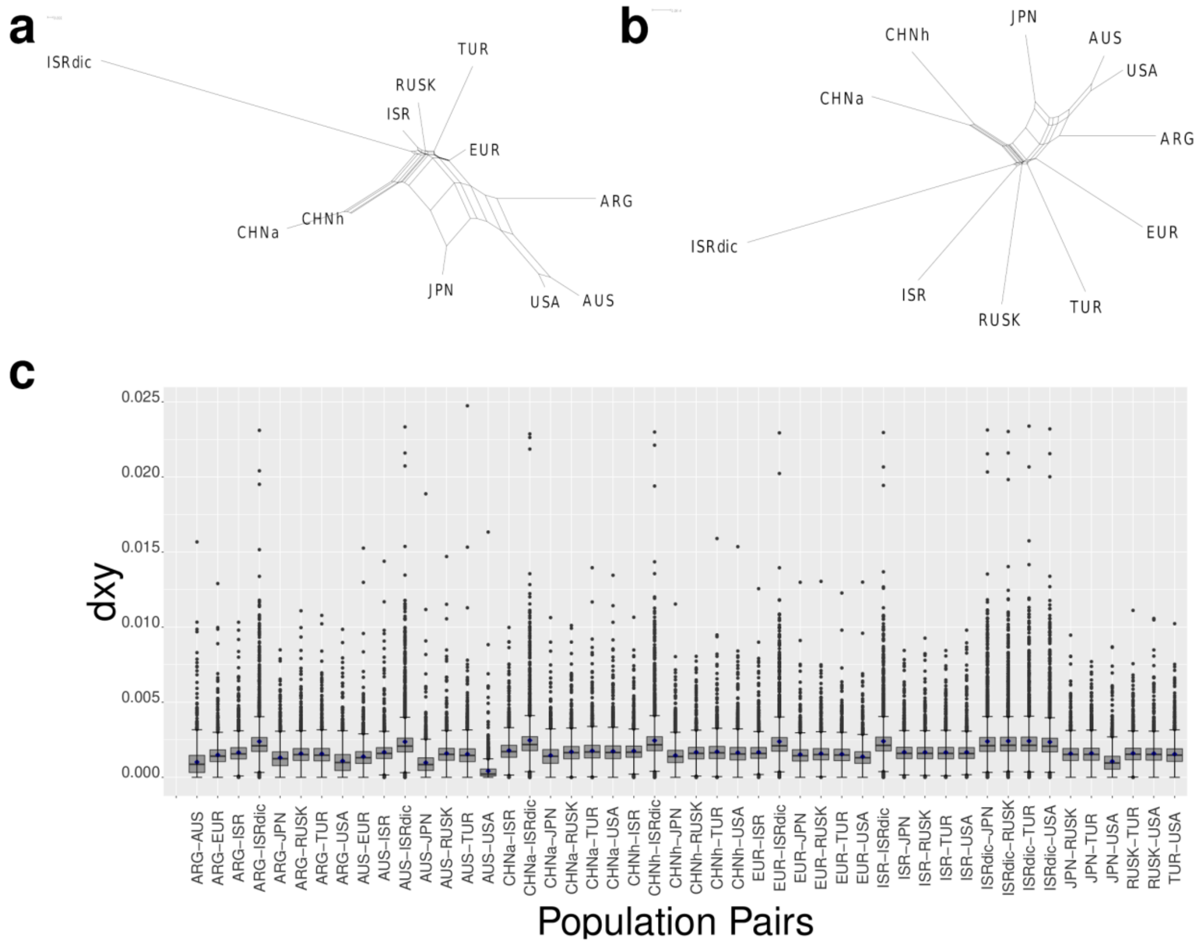

Supplementary Fig. 14. Divergence and diversity distances between populations of the 172 isolates. **(a)** Distance of average differences (DAD) as a phylogenetic network produced with NeighborNet). **(b)** Between populations divergence (dxy) as a phylogenetic network produced with NeighborNet. **(c)** Boxplot graphs of dxy values for all 50kb (n=2,823) genomic windows used for the calculations. Boxes indicate the inter-quartile range (IQR) with the central line indicating the median and whiskers indicating the minimum and maximum without outliers, respectively. Outliers were defined as minimum – 1.5*IQR and maximum + 1.5*IQR, respectively. The average is indicated by a dark blue rhombus. The whiskers indicate the standard error. Source data are provided as a Source Data file.

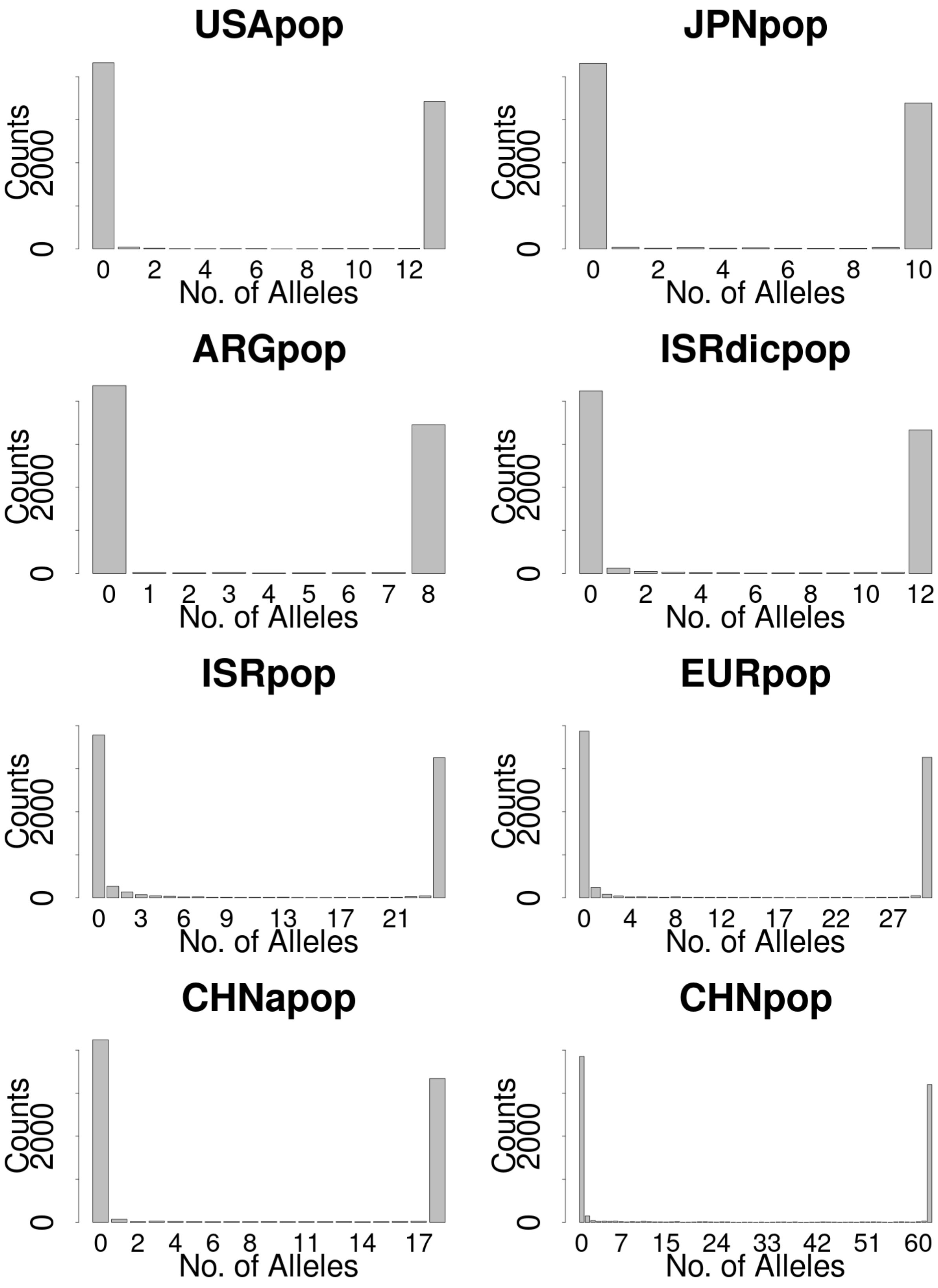

Supplementary Fig. 15. Site Frequency Spectrum/Minor Allele Frequencies graphs. Bar graphs of the minor allele frequency per population. Source data are provided as a Source Data file.

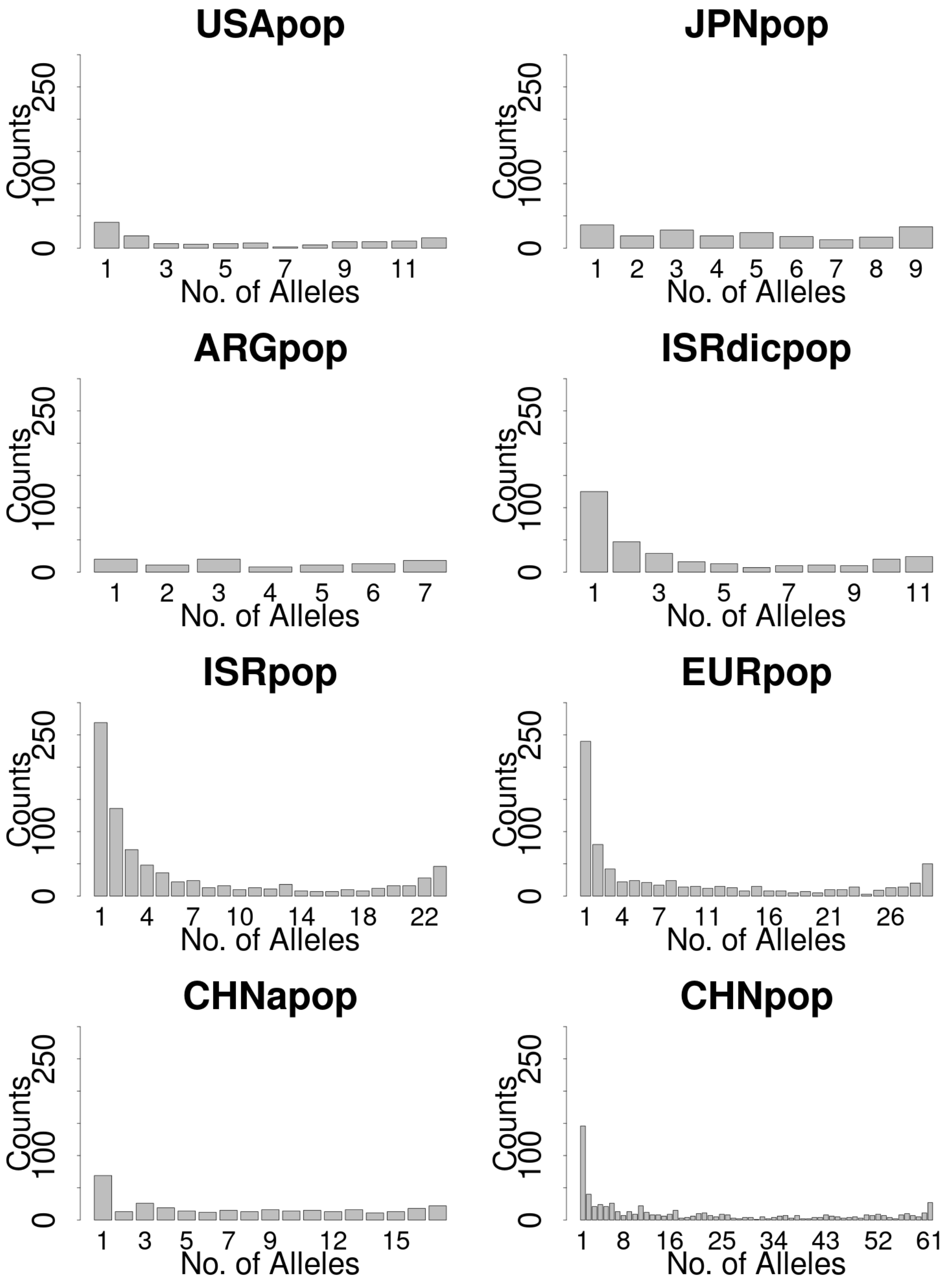

Supplementary Fig. 16. Site Frequency Spectrum/Minor Allele Frequencies graphs version 2. Bar graphs of the minor allele frequency per population excluding alleles with no derived SNPs (No. of Alleles=0) and derived alleles fixed in a population (No. of Alleles=Number of isolates in population).The left peak represents singletons for the ancestral state and the right peak represents singletons for the derived state. For some populations, we see a U-shaped distribution of alleles with two peaks on both sides (e.g. ISRdicpop, ISRpop, EURpop and CHNpop). Source data are provided as a Source Data file.

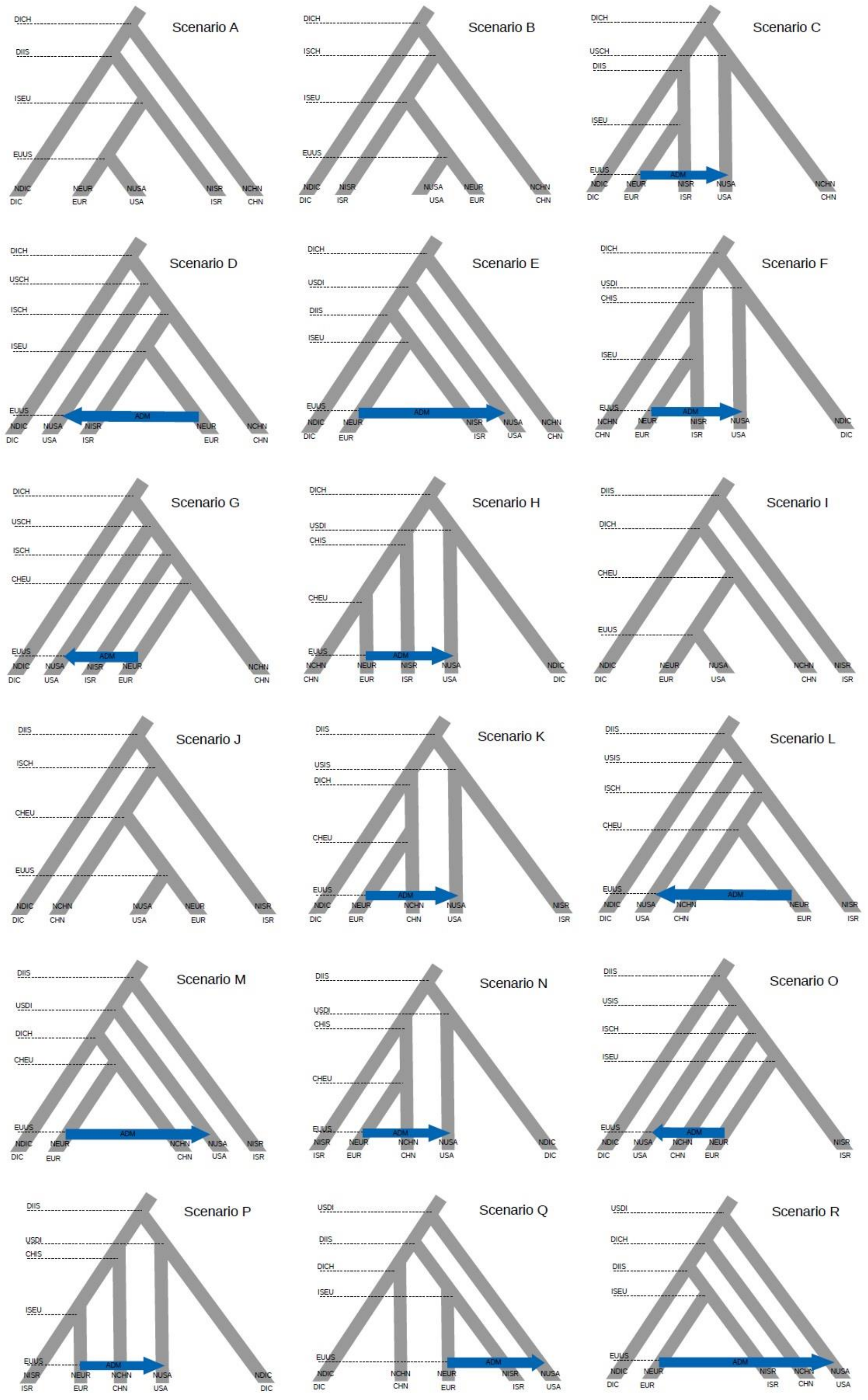

Supplementary Fig. 17. All 25 models used to simulate the history of powdery mildew populations with fastsimcoal2. The various abbreviations refer to the different populations and priors. DIC (*B.g. dicocci*), EUR, ISR, CHN and USA are the five populations used in this analysis, while the abbreviations with the population triplet name and an "N" in front of it represent the effective population size prior (e.g. NUSA is the effective population size prior of the USA mildew population). The ADM abbreviation refers to the possible amount of migration happening from one population to another. Finally, the abbreviations on the left of each scenario scheme refer to the time priors which are explained in the Supplementary Data 3.

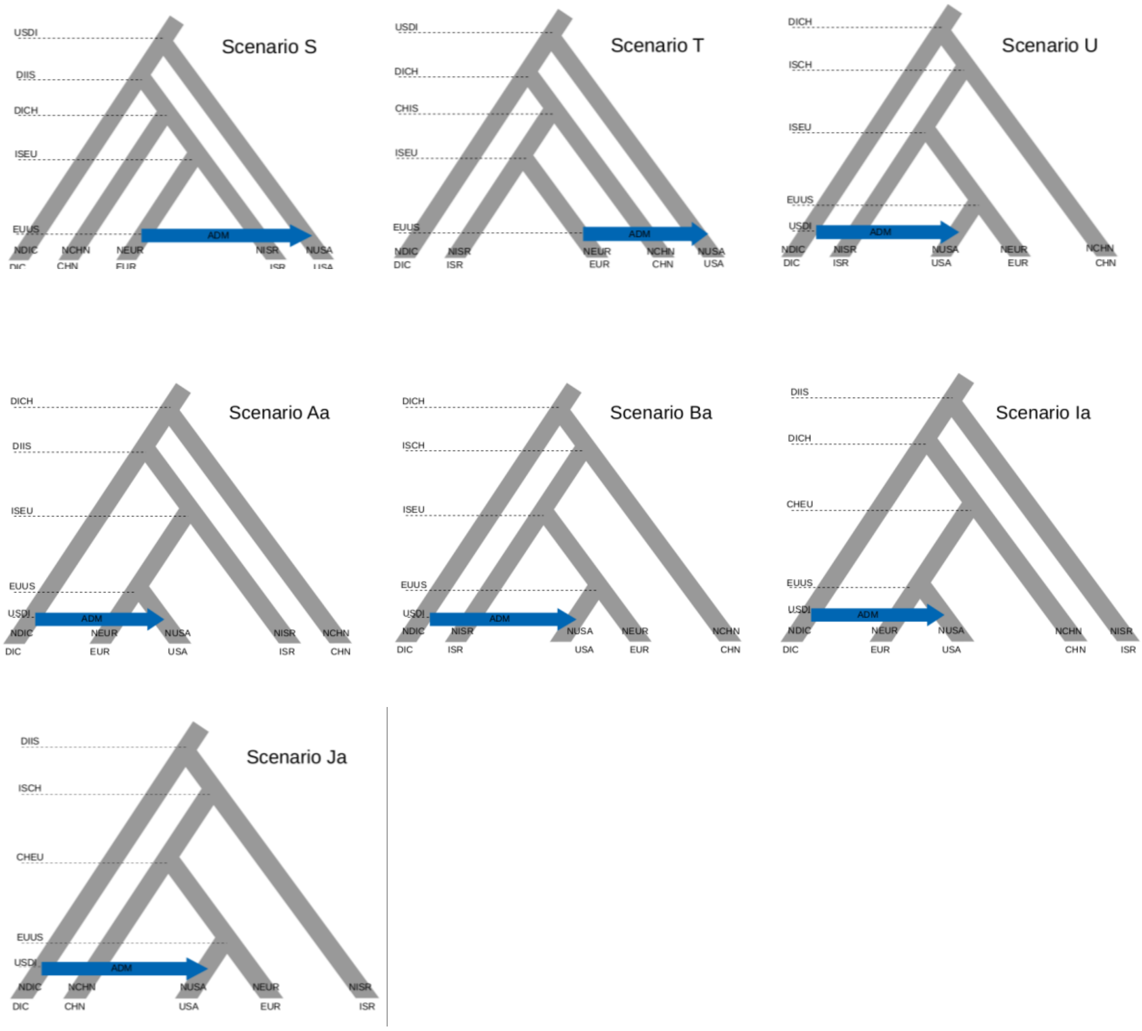

Supplementary Fig. 17 (continued). All 25 models used to simulate the history of powdery mildew populations with fastsimcoal2. The various abbreviations refer to the different populations and priors. DIC (*B.g. dicocci*), EUR, ISR, CHN and USA are the five populations used in this analysis, while the abbreviations with the population triplet name and an "N" in front of it represent the effective population size prior (e.g. NUSA is the effective population size prior of the USA mildew population). The ADM abbreviation refers to the possible amount of migration happening from one population to another. Finally, the abbreviations on the left of each scenario scheme refer to the time priors which are explained in the Supplementary Data 3.

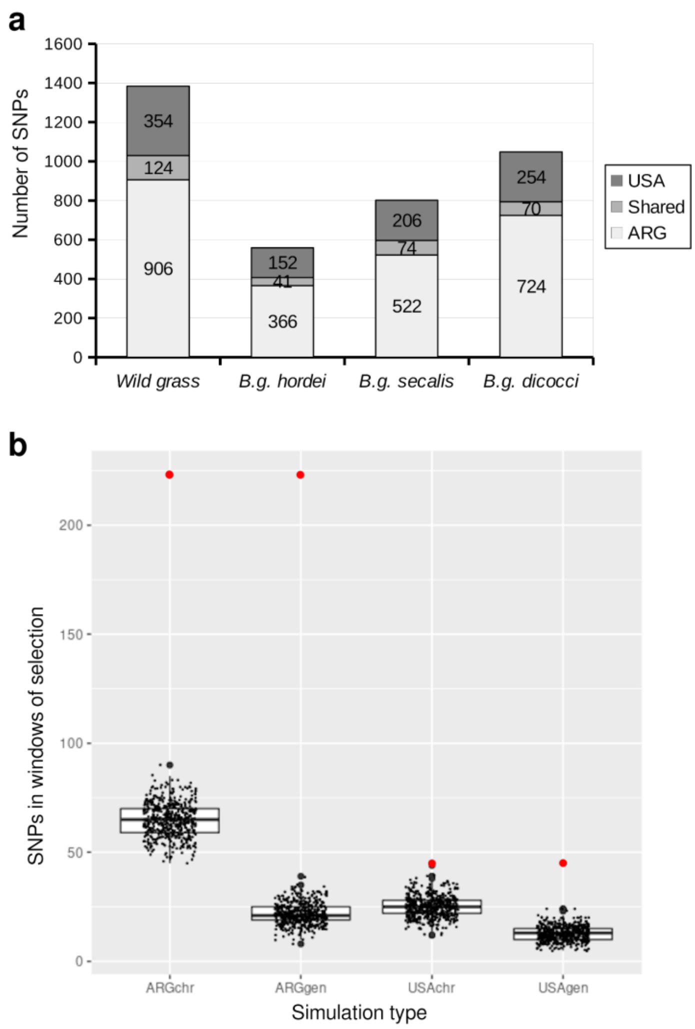

Supplementary Fig. 18. Evidence for ancient hybridizations on mildews from Argentina and the USA. **(a)** Numbers of ancestral SNPs found at near fixation (i.e. in >85% of all isolates) in mildews from Argentina and the USA. The ancestral SNPs were searched in four groups of mildews. The y-axis indicates the number of SNPs that were found in the respective groups. Wild grass mildews include *B.g. lolii*, *B.g. avenae* and *B.g. poae*. **(b)** Results of simulations testing whether the number of SNPs from distantly related mildew donors (ancestral SNPs) in windows that show signature of selection is different from random distributions. The x-axis shows the simulation type. Here, positions of SNPs were randomized in two ways. ARGchr is a simulation where the number of SNPs per chromosome in the mildews from Argentina was kept the same, but their positions on the same chromosome were randomized. ARGgen is a simulation where the total number of SNPs were randomly distributed across the whole genome. USAchr and USAgen were done analogously for the ancestral SNPs found in the mildew population from the USA. Shown are the results of 500 simulations (n=500). Dots indicate the number of SNPs found by chance in a window that showed signature of selection. The red dots show the actual number of SNPs found in the windows showing signatures of selection. Boxes indicate the inter-quartile range (IQR) with the central line indicating the median and whiskers indicating the minimum and maximum without outliers, respectively. Outliers were defined as minimum – 1.5*IQR and maximum + 1.5*IQR, respectively. Windows of selection were highly enriched in mildews from Argentina, and to a lesser degree in mildews from USA. Source data are provided as a Source Data file.

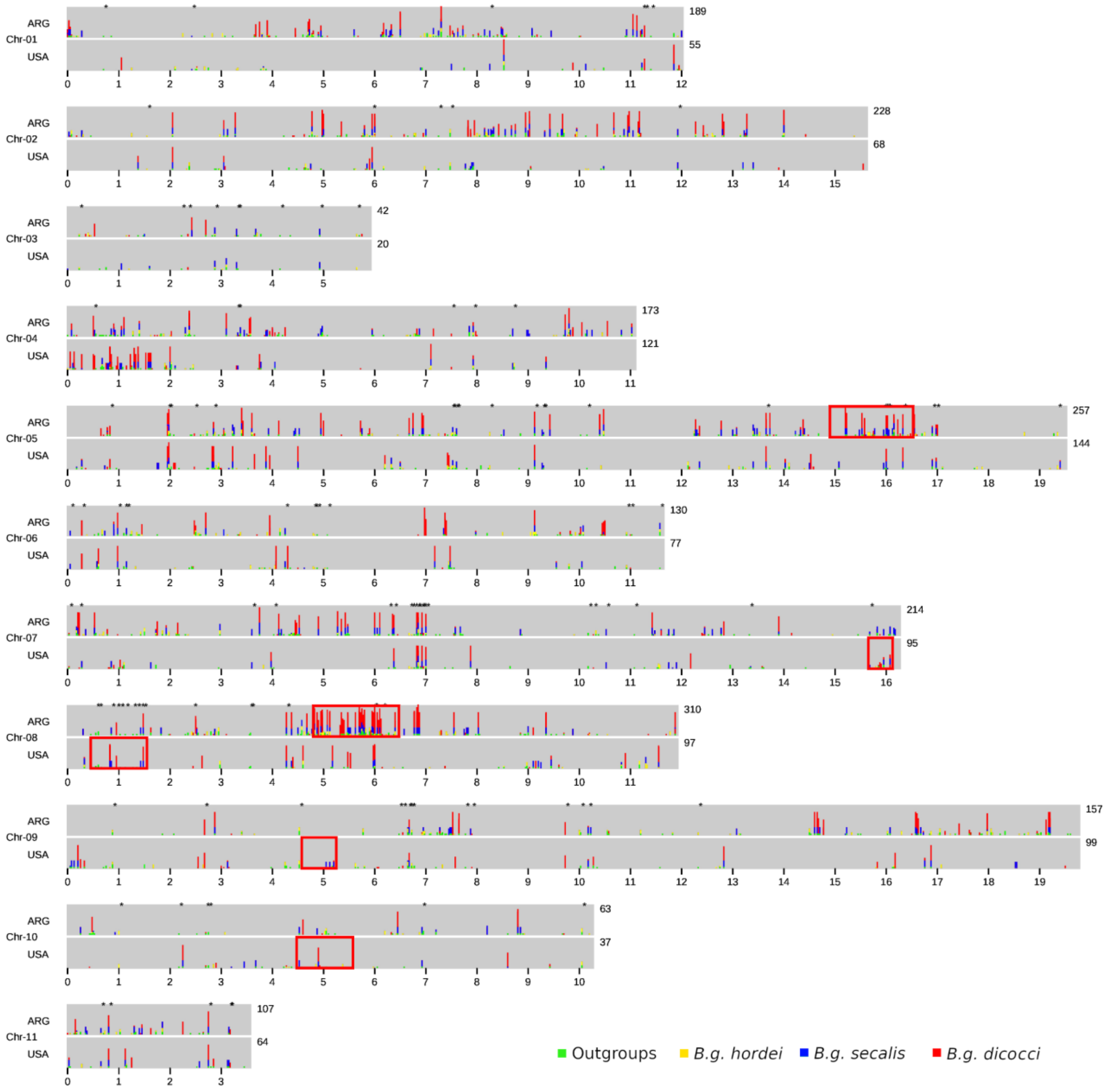

Supplementary Fig. 19. Distribution of ancestral SNPs in the complete USA population along with windows showing signature of selection for all populations. Evidence for ancient hybridizations of mildews in North and South America. Populations from Argentina and USA share numerous SNPs with ancestral mildew lineages. Positions of SNPs are indicated by vertical lines with colors indicating in which ancestral groups the SNPs were found (outgroups: *B.g. lolii*, *B.g. dactylis*, *B.g. avenae*, *B.g. poae*). SNPs shared between populations from Argentina and USA are indicated by asterisks. The red rectangles indicate genomic windows that show signatures of selection.

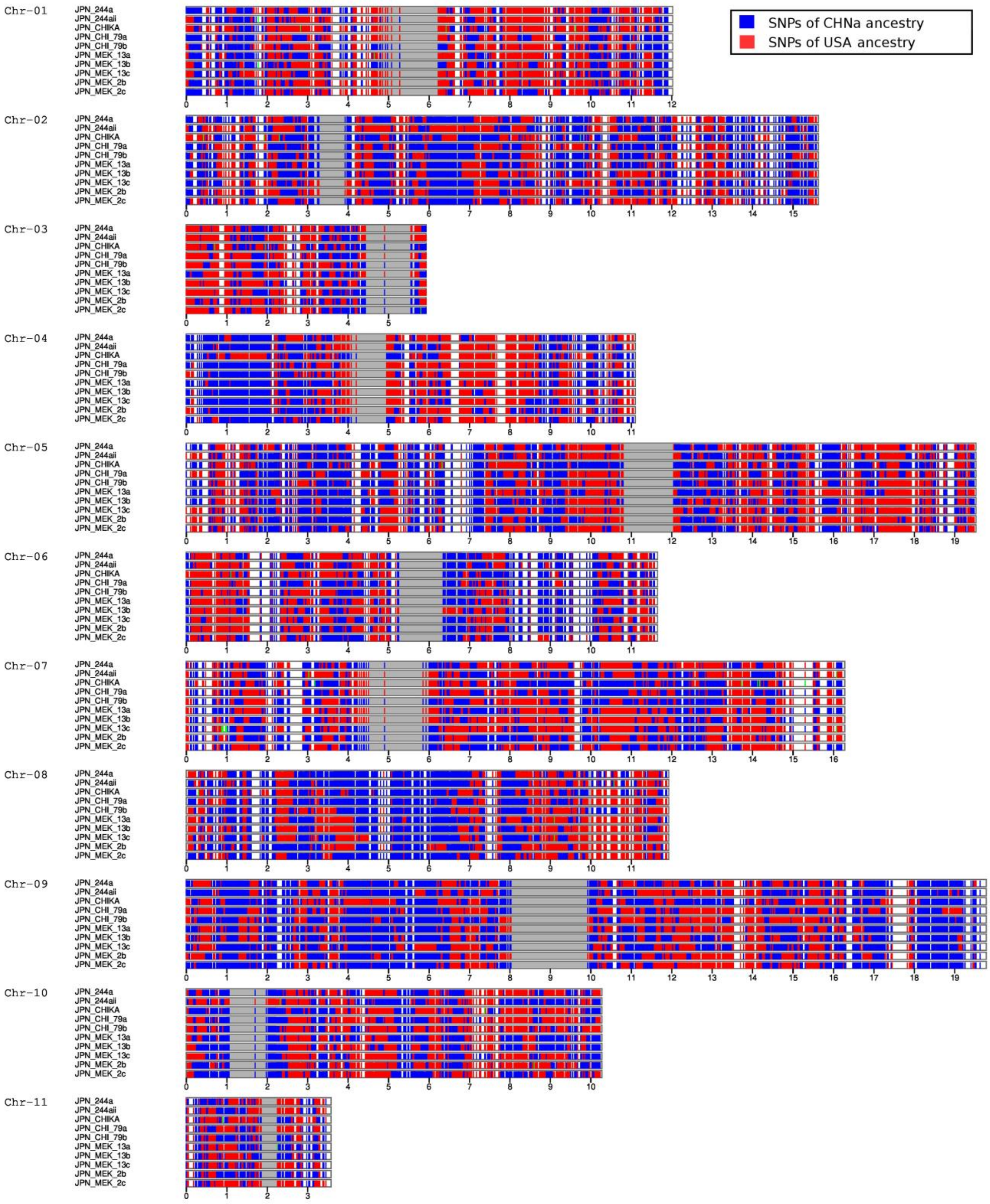

Supplementary Fig. 20. Haploblocks graph of the Japanese isolates using the USA and the CHNa isolates as parental haplotypes for the whole genome. The CHNa haplotypes are depicted in dark blue and the USA haplotypes are depicted in red. Centromere positions are filled with the colour grey.

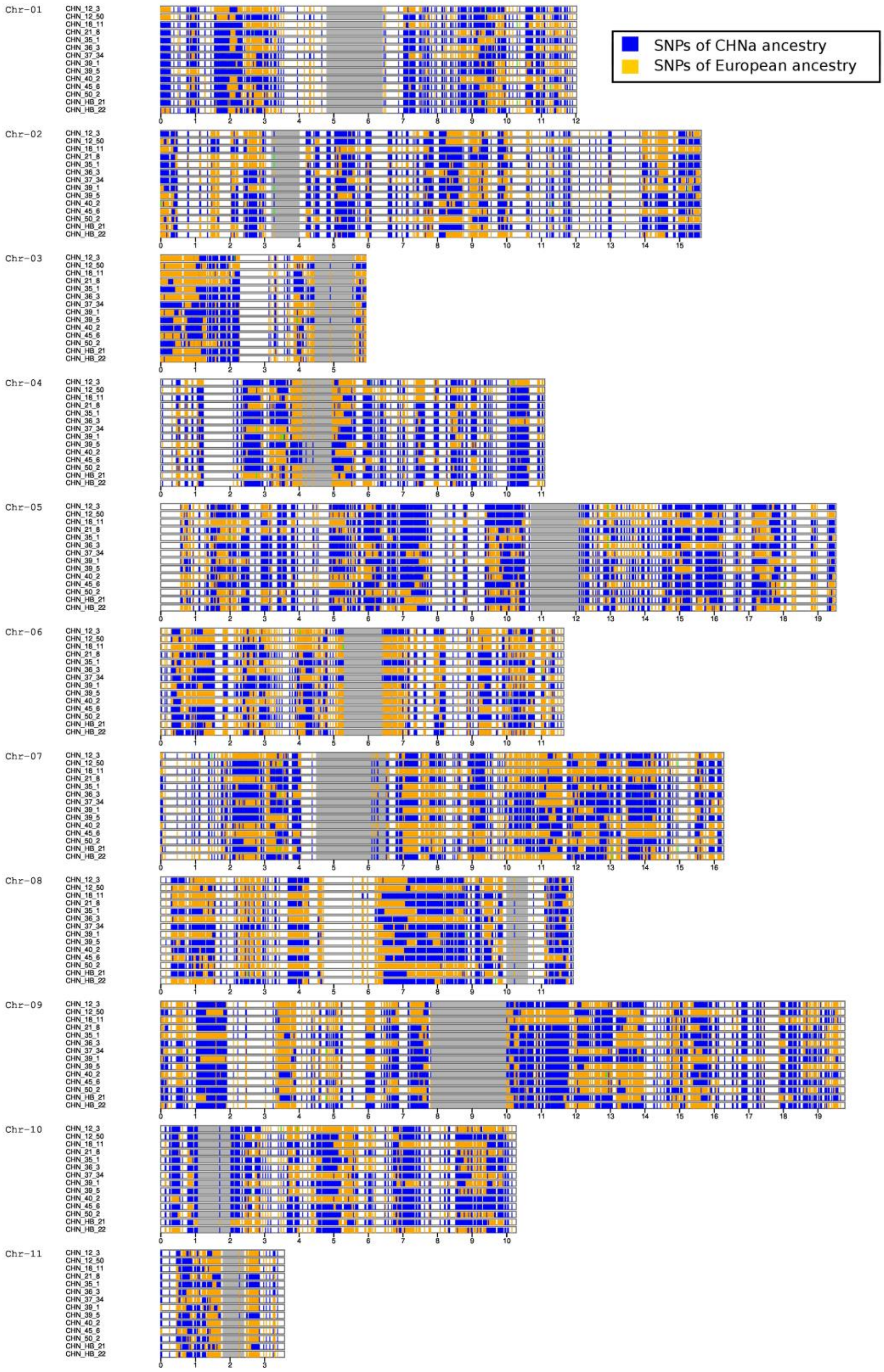

Supplementary Fig. 21. Haploblocks graph of the CHNh isolates using the EUR and the CHNa isolates as parental haplotypes for the whole genome. The CHNa haplotypes are depicted in dark blue and the EUR haplotypes are depicted in yellow. Centromere positions are filled with the colour grey.

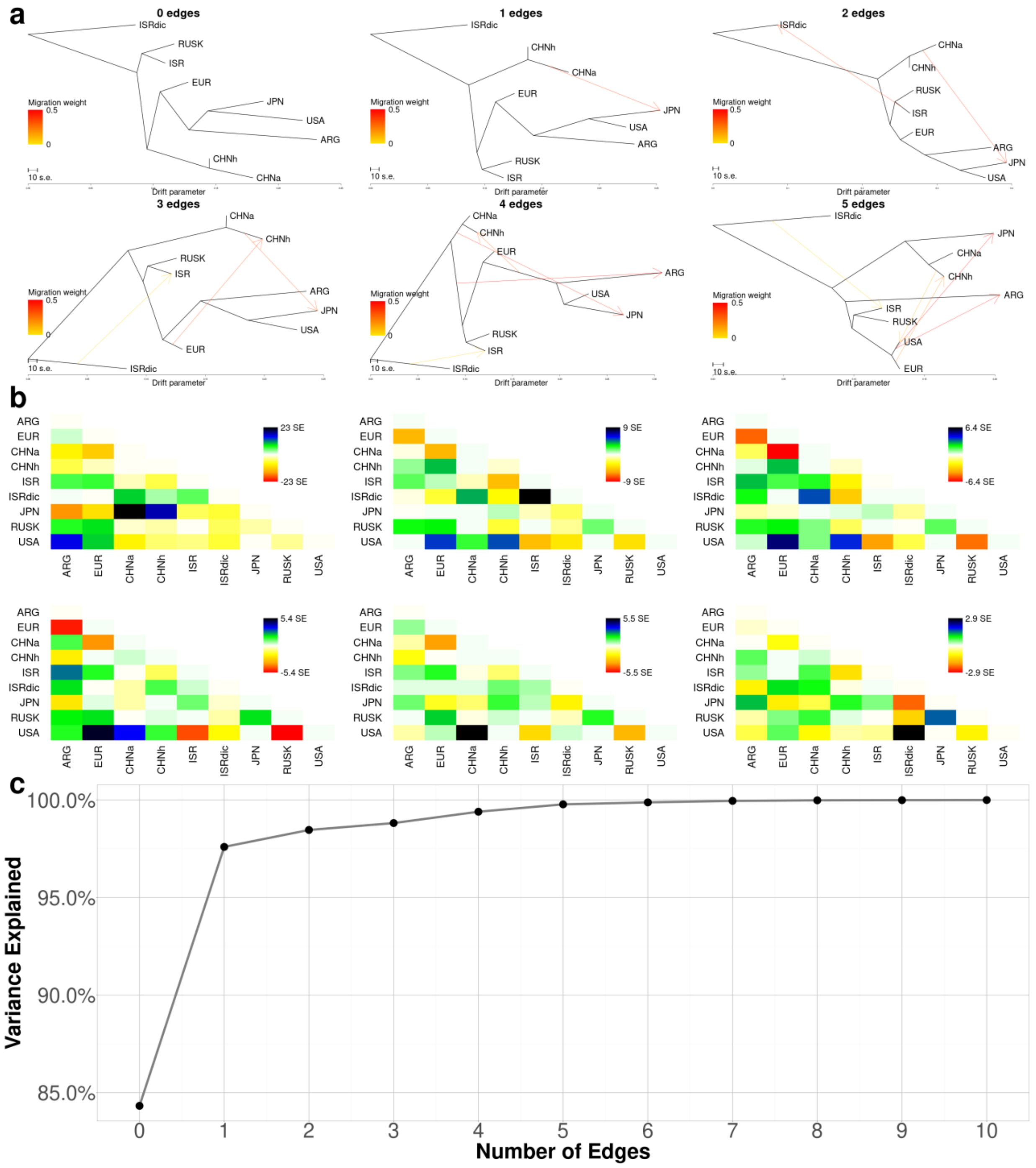

Supplementary Fig. 22. TreeMix analysis, a method for inferring the patterns of population splits and mixtures in the history of a set of populations, using SNPs and the parameters: bs, k500, seed12345. There were various tree analyses with zero to 20 migration events. *B.g. dicocci* was always used as an outgroup for these analyses. **(a)** Maximum likelihood trees with arrows depicting the various migrations/edges (the first five). The more intense the red colour is, the closer to 50% of migration happening. **(b)** The residual fit from the maximum likelihood tree for zero to five migration events (from left to right). The colours are described in the palette on the right of each plot. Residuals above zero represent populations that are more closely related to each other in the data than in the best-fit tree, and thus are candidates for admixture events. **(c)** Percentage of the variance explained by the different number of migration events. We see most of the variance is explained with one edge/migration event. Source data are provided as a Source Data file.

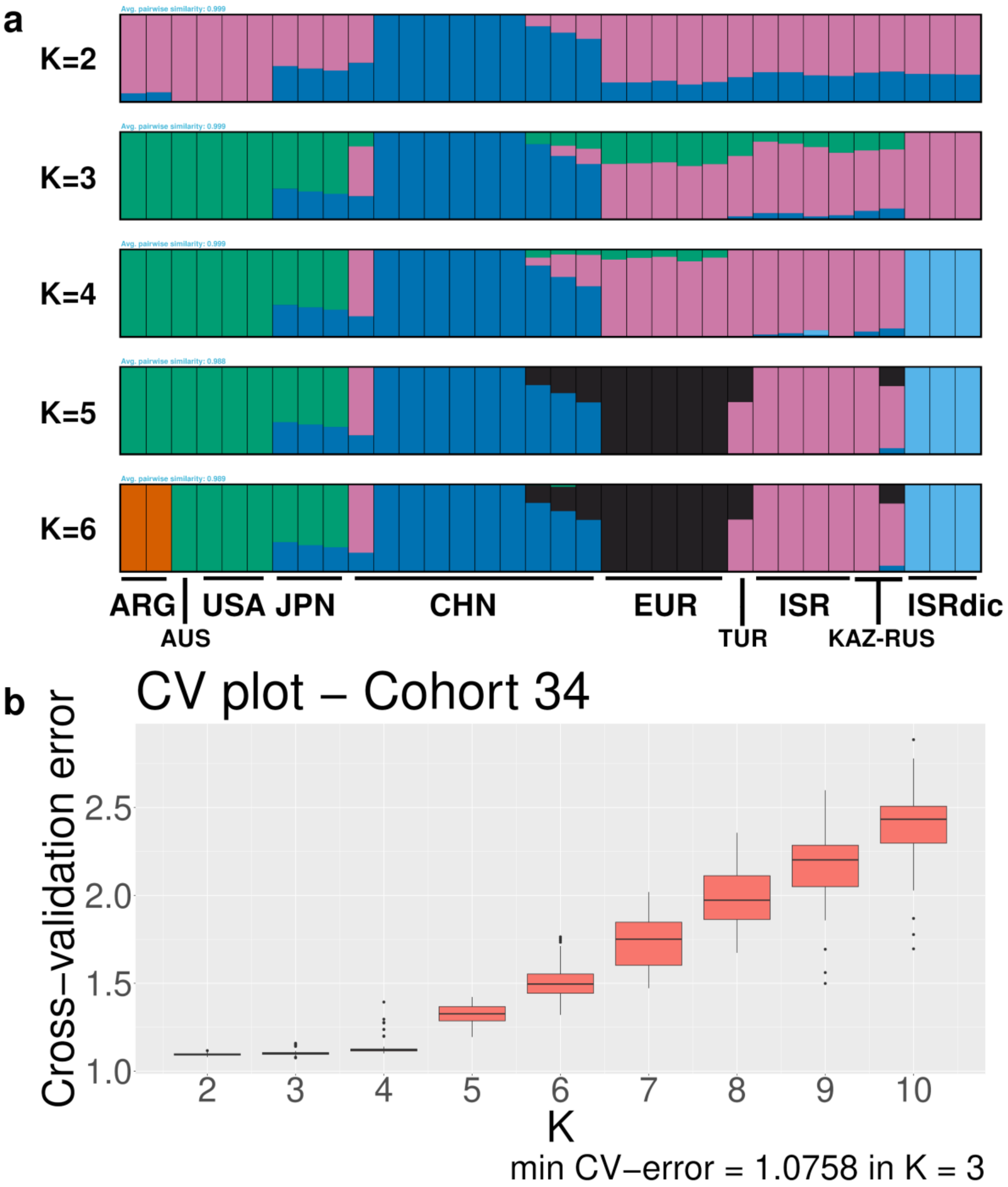

Supplementary Fig. 23. (a) Results of the sub-sampled (34 isolates) ADMIXTURE analysis (for up to K=6) and **(b)** its CV plot (for up to K=10) (n=100). Boxes indicate the inter-quartile range (IQR) with the central line indicating the median and whiskers indicating the minimum and maximum without outliers, respectively. Outliers were defined as minimum – 1.5*IQR and maximum + 1.5*IQR, respectively. Source data are provided as a Source Data file.

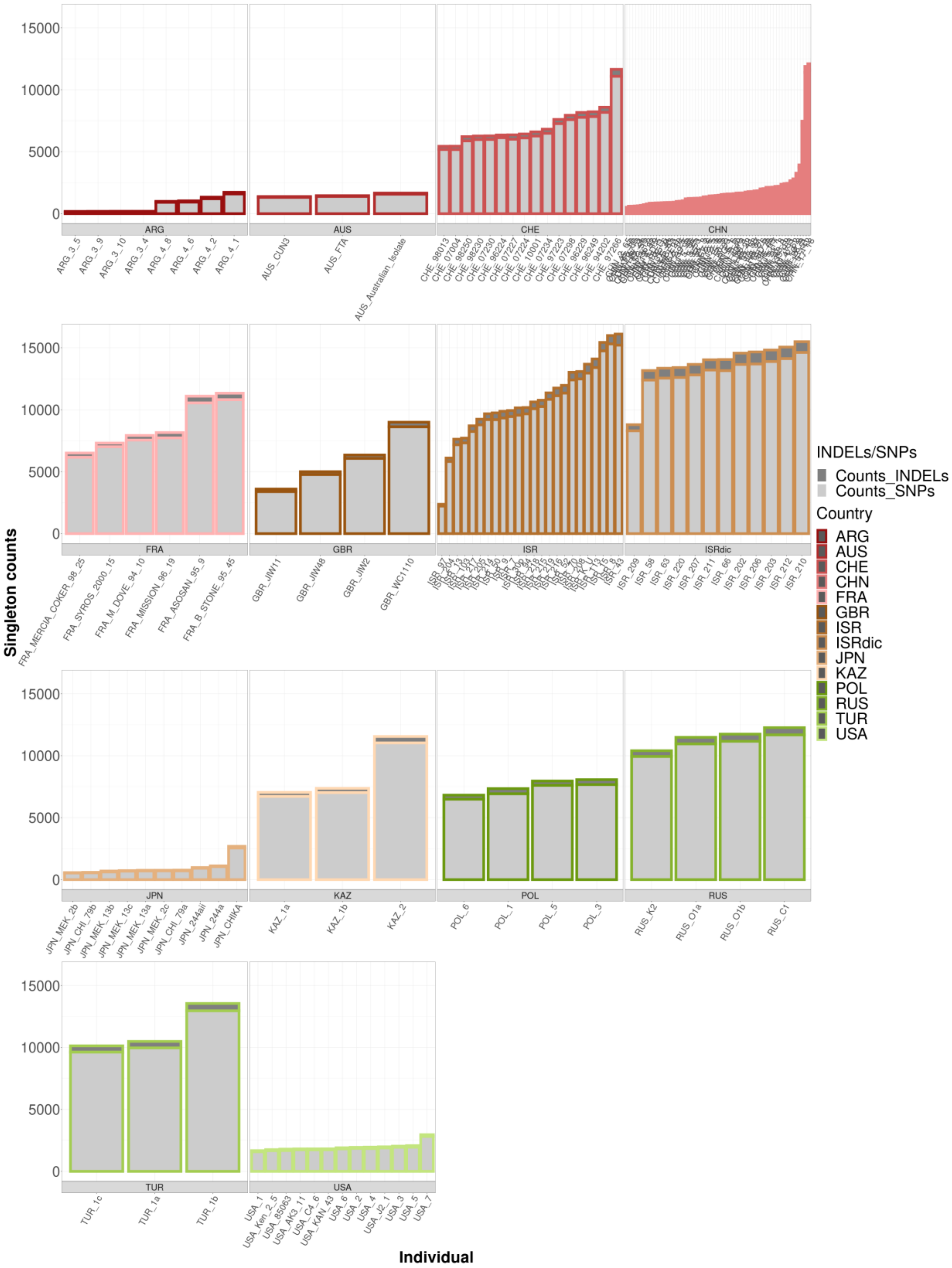

Supplementary Fig. 24. Singleton SNPs from the 172 non-clonal samples within the different populations using the whole dataset. In dark gray are the INDELs and in light gray the SNPs that constitute the majority of the data. Source data are provided as a Source Data file.

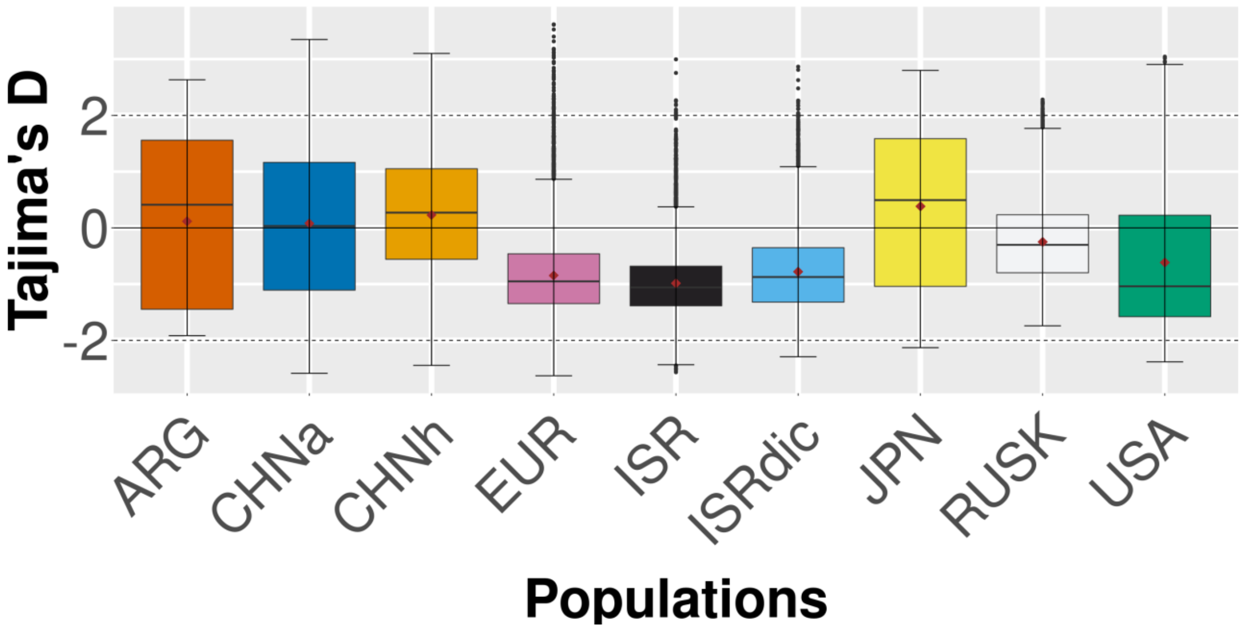

Supplementary Fig. 25. Tajima's D in 10kb windows (n= 13,718) for various populations. Boxes indicate the inter-quartile range (IQR) with the central line indicating the median and whiskers indicating the minimum and maximum without outliers, respectively. Outliers were defined as minimum – 1.5*IQR and maximum + 1.5*IQR, respectively. The average is indicated by the brown rhombus. Source data are provided as a Source Data file.

Supplementary Tables

Supplementary Table 1. Summary table of all the isolates per country of origin. The number of isolates with a specific mating type (MAT1=MAT-1-1-3, MAT2=MAT1-2-1), *forma specialis* and how many were used after removing the clonal, cross-contaminated and very low coverage isolates. The mating types were identified by blasting the mating type loci on de novo genome assemblies of the isolates.

Country	Code	Isolates	MAT1	MAT2	Fsp. tritici	Fsp. dicocci	Used
**Argentina**	ARG	8	3	5	8	0	8
**Australia**	AUS	3	3	0	3	0	3
**China**	CHN	100	40[a]	63[a]	100	0	62
**France**	FRA	7	2	5	7	0	6
**Israel**	ISR	46	21	25	34	12	37
**Japan**	JPN	11	3	8	11	0	10
**Kazakhstan**	KAZ	3	1	2	3	0	3
**Poland**	POL	4	4	0	4	0	4
**Russia**	RUS	4	3	1	4	0	4
**Switzerland**	CHE	16	7	9	16	0	16
**Turkey**	TUR	3	0	3	3	0	3
**UK**	GBR	5	2	3	5	0	5
**USA**	USA	14	6	8	14	0	13
**SUM**		224	95	132	212	12	172

[a]The sum of MAT1 and MAT2 can be higher than the total number of isolates for the Chinese mildew population, because some samples had both mating types because they were possibly cross-contaminated and removed from the analyses (see methods).

Supplementary Table 2. Summary table of all the isolates' mating type and a statistical test (two-sided) to check if the populations deviate from the expected 50/50 ratio. The number of isolates with a specific mating type is signified by: MAT1=MAT-1-1-3, MAT2=MAT1-2-1. The mating types were identified by blasting the mating type loci on de novo genome assemblies of the isolates. The chi-squared test was performed only for the populations/groups that had a sample size larger than 50. Moreover, only the datasets with isolates more than five isolates where considered for the Fisher's exact test.

Dataset	MAT1	MAT2	Fisher's Exact Test (p-value)	Chi-squared Test (value)	Chi-squared Test (p-value)
ALL (172 isolates)	78	94	0.4499	X-squared = 0.571	p-value = 0.4499
ALL (no *B.g. dicocci*)	72	88	0.4333	X-squared = 0.61404	p-value = 0.4333
ARG	4	4	1.0000	Not applicable	Not applicable
CHE	7	9	1.0000	Not applicable	Not applicable
CHN	39	23	0.2047	X-squared = 1.6074	p-value = 0.2049
EUR	15	15	1.0000	Not applicable	Not applicable
ISR	14	10	0.7725	Not applicable	Not applicable
ISRdic	6	6	1.0000	Not applicable	Not applicable
JPN	3	7	0.6499	Not applicable	Not applicable
USA	6	7	1.0000	Not applicable	Not applicable

Supplementary Table 3. Nucleotide Diversity and Watterson's Theta for the eight populations and for different regions of the genome (whole genome, intergenic regions, genes, exons, introns). The whole genome calculation was performed in windows of 10kb. For the LD-pruned and unfiltered SNPs, ADW was calculated.

Pi	Whole Genome	Intergenic Regions	Genes	Exons	Introns	LD-pruned SNPs	Unfiltered SNPs
ARG	0.000283	0.000282	0.000184	0.000160	0.000234	0.077000	0.018000
EUR	0.000794	0.000935	0.000619	0.000521	0.000781	0.276000	0.067000
CHNh	0.000569	0.000860	0.000576	0.000496	0.000740	0.134000	0.040000
CHNa	0.000434	0.000594	0.000395	0.000350	0.000516	0.210000	0.057000
ISR	0.000978	0.001143	0.000739	0.000639	0.000946	0.301000	0.080000
ISRdic	0.000636	0.000655	0.000441	0.000385	0.000543	0.050000	0.044000
JPN	0.000390	0.000472	0.000317	0.000273	0.000406	0.118000	0.031000
USA	0.000357	0.000260	0.000162	0.000146	0.000220	0.069000	0.017000
Average	0.000477	0.000567	0.000373	0.000322	0.000477	0.140401	0.038669

Theta	Whole Genome	Intergenic Regions	Genes	Exons	Introns
ARG	0.000272	0.000286	0.000205	0.000210	0.000463
EUR	0.001212	0.001293	0.000937	0.000966	0.002162
CHNh	0.000583	0.000873	0.000654	0.000668	0.001520
CHNa	0.000468	0.000611	0.000462	0.000481	0.001088
ISR	0.001396	0.001628	0.001172	0.001223	0.002728
ISRdic	0.000808	0.000848	0.000624	0.000666	0.001387
JPN	0.000387	0.000459	0.000339	0.000354	0.000773
USA	0.000401	0.000303	0.000217	0.000231	0.000501
Average	0.000627	0.000716	0.000522	0.000543	0.001207

Supplementary Table 4. F4 statistics for the different combinations of populations to look at the shared ancestry between them. *B.g. poae* is used as the ougroup. CHNa refers to the mildew isolates from China that have 99.9% of the ancestry from East Asia. Only the significant results with Z>3 are depicted here.

W	X	Y	Z	f4	stderr	Zscore	BABA	ABBA	nsnps
ISR	EUR	ISRdic	BGPOA	1.82E-03	1.25E-04	14.499	1189	817	204687
CHNa	EUR	ISRdic	BGPOA	0.002038	1.48E-04	13.756	1279	862	204687
CHN	EUR	ISRdic	BGPOA	0.001341	1.05E-04	12.739	1125	850	204687
CHNh	EUR	ISRdic	BGPOA	1.57E-03	1.34E-04	11.71	1158	838	204687
RUSK	EUR	ISRdic	BGPOA	1.36E-03	1.34E-04	10.176	1091	812	204687
JPN	EUR	ISRdic	BGPOA	1.33E-03	1.58E-04	8.414	1072	800	204687
ARG	EUR	ISRdic	BGPOA	0.00117	1.52E-04	7.713	1046	806	204680
USA	EUR	ISRdic	BGPOA	0.001154	1.64E-04	7.032	972	736	204687
CHNa	CHN	ISRdic	BGPOA	6.96E-04	1.01E-04	6.916	714	572	204687
ISR	RUSK	ISRdic	BGPOA	4.57E-04	8.70E-05	5.252	959	866	204687
CHNa	JPN	ISRdic	BGPOA	7.11E-04	1.38E-04	5.135	911	766	204687
CHNa	ARG	ISRdic	BGPOA	8.67E-04	1.69E-04	5.122	1091	914	204680
CHNa	USA	ISRdic	BGPOA	8.84E-04	1.94E-04	4.562	1125	945	204687
CHNa	RUSK	ISRdic	BGPOA	6.76E-04	1.58E-04	4.285	1010	871	204687
ISR	ARG	ISRdic	BGPOA	6.48E-04	1.59E-04	4.08	1017	885	204680
ISR	CHN	ISRdic	BGPOA	4.77E-04	1.27E-04	3.76	1032	935	204687
CHNa	CHNh	ISRdic	BGPOA	4.73E-04	1.27E-04	3.713	700	603	204687
ISR	USA	ISRdic	BGPOA	6.64E-04	1.79E-04	3.702	1026	890	204687
ISR	JPN	ISRdic	BGPOA	4.92E-04	1.61E-04	3.056	992	891	204687

Supplementary Table 5. The results from the fastsimcoal analysis of all 25 models used to simulate the history of powdery mildew populations with fastsimcoal2 using the five populations (ISRdic, CHNa, EUR, USA, ISR). The top three highest probability scenarios are in green font.

Scenarios	average*	min*	max*	deltaL	AIC
ScenarioA	-41673.5	-41704	-41641	19260.392	191788
ScenarioAa[1]	-40432.6	-40482	-40391	18009.877	186032
ScenarioB	-42503.9	-42533	-42460	20079.431	195560
ScenarioBa	-41089.7	-41140	-41043	18662.384	189037
ScenarioC	-43176.8	-43223	-43130	20749.371	198647
ScenarioD	-43441.1	-43476	-43405	21024.232	199913
ScenarioE	-42354.5	-42396	-42309	19928.644	194868
ScenarioF	-43219.3	-43262	-43184	20803.294	198896
ScenarioG	-43172.8	-43287	-42969	20588.648	197907
ScenarioH	-43079.5	-43122	-43040	20659.484	198234
ScenarioI	-43019.2	-43054	-42968	20587.12	197898
ScenarioIa[1]	-35008.4	-35121	-34757	12376.111	160087
ScenarioJ	-43140	-43397	-43065	20683.895	198344
ScenarioJa[1]	-35370.8	-35476	-35173	12792.727	162006
ScenarioK	-44031.4	-44109	-43949	21568.347	202419
ScenarioL	-43871.5	-43921	-43829	21447.883	201864
ScenarioM	-43372	-43488	-43249	20868.248	199195
ScenarioN	-43111.1	-43180	-43050	20669.444	198279
ScenarioO	-42642.7	-42740	-42506	20124.978	195772
ScenarioP	-43347.9	-43414	-43285	20904.513	199362
ScenarioQ	-43983.8	-44040	-43910	21528.89	202237
ScenarioR	-43600.1	-43666	-43541	21160.649	200541
ScenarioS	-44098.8	-44156	-44049	21668.769	202881
ScenarioT	-44520.1	-44575	-44465	22084.021	204794
ScenarioU	-41091	-41131	-41055	18674.614	189093

*Referring to the Maximum Likelihood result

[1]Top three highest probability scenarios

Supplementary Table 6. F3 statistics for the JPN population using different populations as parents. The best supported pair of parents, even though non-significant, is where Z is closest to Z=-3, when CHNa and USA are placed as the parents of the JPN population.

A	B	C	f3	stderr	Zscore	nsnps
**CHNa**	**USA**	**JPN**	**0.117523**	**0.028165**	**4.173**	**332626**
CHNh	USA	JPN	0.195275	0.031465	6.206	357579
CHN	USA	JPN	0.241372	0.03382	7.137	387827
RUSK	USA	JPN	0.355409	0.038463	9.24	393637
ISR	USA	JPN	0.364581	0.039373	9.26	679311
ISRdic	USA	JPN	0.383517	0.03935	9.746	476874
EUR	USA	JPN	0.477463	0.045677	10.453	584193
ARG	USA	JPN	0.54752	0.04883	11.213	242618
ARG	CHNa	JPN	0.33092	0.025924	12.765	326044
ARG	CHNh	JPN	0.381793	0.029534	12.927	351014
ARG	CHN	JPN	0.420965	0.032123	13.105	381850
ARG	EUR	JPN	0.586268	0.039838	14.716	580088
ARG	ISRdic	JPN	0.558108	0.036872	15.136	471658
ARG	RUSK	JPN	0.552694	0.036464	15.157	383707
ARG	ISR	JPN	0.570776	0.036137	15.795	667850
CHNa	EUR	JPN	0.478917	0.030139	15.89	653306
CHNa	ISRdic	JPN	0.611891	0.038437	15.919	543291
CHNa	CHNh	JPN	0.917774	0.056624	16.208	362113
CHNh	ISRdic	JPN	0.621427	0.038145	16.291	571176
CHNh	EUR	JPN	0.577527	0.035396	16.316	641314
CHNh	ISR	JPN	0.596412	0.034023	17.53	738402
CHNa	ISR	JPN	0.57529	0.032596	17.649	736314
CHN	ISRdic	JPN	0.652648	0.036299	17.98	599390
CHN	EUR	JPN	0.62563	0.034722	18.018	667366
CHNh	RUSK	JPN	0.598709	0.033114	18.08	468785
ISR	ISRdic	JPN	0.824844	0.04549	18.132	886985
CHNa	RUSK	JPN	0.576305	0.031696	18.182	457156
EUR	ISRdic	JPN	0.72416	0.039736	18.224	804620
EUR	ISR	JPN	0.727213	0.039363	18.475	889686
CHN	ISR	JPN	0.630515	0.034122	18.478	764868
CHN	CHNa	JPN	0.95931	0.051551	18.609	384430
ISRdic	RUSK	JPN	0.751006	0.040108	18.724	609948
CHN	CHNh	JPN	0.859732	0.045853	18.75	386836
EUR	RUSK	JPN	0.722824	0.037584	19.232	660451
ISR	RUSK	JPN	0.777656	0.039285	19.795	737380
CHN	RUSK	JPN	0.635536	0.031926	19.907	497430

Supplementary Table 7. F4 statistics for the different combinations of populations to check which populations share most with the JPN mildew population. *B.g. poae* is used as the ougroup. CHNa refers to the mildew isolates from China that have 99.9% of the ancestry from East Asia. Only the significant results are shown with Z>3 and the ones with Z<-3.

W	X	Y	Z	f4	stderr	Zscore	BABA	ABBA	nsnps
JPN	CHNh	USA	BGPOA	0.007059	0.00035	20.158	2145	701	204687
JPN	CHN	USA	BGPOA	0.006935	0.000357	19.414	2168	748	204687
JPN	CHNa	USA	BGPOA	0.007696	0.000405	18.989	2176	600	204687
JPN	ISR	USA	BGPOA	0.007272	0.000403	18.05	2311	823	204687
JPN	RUSK	USA	BGPOA	0.006853	0.000397	17.276	2233	830	204687
JPN	EUR	USA	BGPOA	0.005644	0.000385	14.661	2174	1019	204687
JPN	EUR	CHNa	BGPOA	0.005981	0.000429	13.935	2148	924	204687
JPN	EUR	CHNh	BGPOA	0.004274	0.000313	13.642	1948	1074	204687
JPN	USA	CHNa	BGPOA	0.005112	0.000395	12.951	1647	600	204687
JPN	EUR	CHN	BGPOA	0.003423	0.000267	12.818	1823	1123	204687
JPN	USA	CHNh	BGPOA	0.003777	0.000299	12.635	1474	701	204687
JPN	ARG	CHNa	BGPOA	0.004125	0.000356	11.588	1746	902	204680
JPN	ARG	CHNh	BGPOA	0.003302	0.000289	11.42	1614	938	204680
JPN	ISR	CHN	BGPOA	0.002968	0.000274	10.828	1775	1167	204687
JPN	EUR	ARG	BGPOA	0.003835	0.000356	10.787	1863	1078	204680
JPN	ARG	CHN	BGPOA	0.002596	0.000247	10.521	1497	966	204680
JPN	ISR	CHNh	BGPOA	0.003574	0.000342	10.461	1877	1146	204687
JPN	CHN	ARG	BGPOA	0.003813	0.000365	10.433	1746	966	204680
JPN	CHNh	ARG	BGPOA	0.003768	0.000372	10.129	1709	938	204680
JPN	ISR	CHNa	BGPOA	0.003939	0.000412	9.551	1966	1159	204687
JPN	ISR	ARG	BGPOA	0.003756	0.000395	9.511	1871	1102	204680
JPN	RUSK	ARG	BGPOA	0.003348	0.000361	9.27	1794	1109	204680
JPN	USA	CHN	BGPOA	0.002902	0.000316	9.17	1342	748	204687
JPN	CHNa	ARG	BGPOA	0.003894	0.000436	8.923	1699	902	204680
JPN	RUSK	CHNh	BGPOA	0.002774	0.000349	7.947	1764	1197	204687
JPN	RUSK	CHN	BGPOA	0.002161	0.000278	7.767	1662	1220	204687
JPN	RUSK	CHNa	BGPOA	0.003117	0.000443	7.035	1852	1214	204687
JPN	CHNa	EUR	BGPOA	0.001525	0.000235	6.482	1236	924	204687
JPN	EUR	ISR	BGPOA	0.00128	0.000214	5.98	1421	1159	204687
JPN	EUR	RUSK	BGPOA	0.001451	0.00026	5.578	1486	1189	204687
JPN	ISR	EUR	BGPOA	0.000939	0.000201	4.665	1351	1159	204687
JPN	USA	RUSK	BGPOA	0.00091	0.000226	4.03	1016	830	204687
JPN	USA	ISR	BGPOA	0.000572	0.000172	3.319	940	823	204687
JPN	RUSK	ISR	BGPOA	-0.000812	0.000203	-4.008	1223	1390	204687
JPN	USA	EUR	BGPOA	-0.001396	0.000213	-6.549	733	1019	204687
JPN	USA	ARG	BGPOA	-0.00258	0.000354	-7.279	818	1347	204680
JPN	CHNh	CHNa	BGPOA	-0.005153	0.000615	-8.376	1147	2201	204687
JPN	CHN	CHNh	BGPOA	-0.003476	0.000379	-9.165	1246	1957	204687
JPN	CHN	CHNa	BGPOA	-0.00521	0.000526	-9.908	1200	2267	204687
JPN	CHNa	CHNh	BGPOA	-0.005849	0.000487	-12.019	1004	2201	204687
JPN	CHNh	CHN	BGPOA	-0.004227	0.000346	-12.202	1092	1957	204687
JPN	CHNa	CHN	BGPOA	-0.006658	0.000436	-15.279	904	2267	204687

Supplementary Table 8. F3 statistics for the CHNh (Chinese recombinant isolates) population using different populations as parents. Only the significant results with Z<-3 are depicted, with the best result being CHNa and EUR as the parent of the recombinant CHNh population.

Non LD-pruned SNPs

A	B	C	f3	stderr	Zscore	nsnps
**CHNa**	**EUR**	**CHNh**	**-0.054896**	**0.004582**	**-11.98**	**664580**
CHNa	USA	CHNh	-0.043393	0.007356	-5.899	388805
CHNa	ARG	CHNh	-0.028677	0.006101	-4.7	374681

LD-pruned SNPs

A	B	C	f3	stderr	Zscore	nsnps
**CHNa**	**EUR**	**CHNh**	**-0.045621**	**0.003989**	**-11.435**	**317599**
CHNa	USA	CHNh	-0.033191	0.006773	-4.901	162893
CHNa	ARG	CHNh	-0.018815	0.006105	-3.082	151722

Supplementary Table 9. List of the isolates used for the sub-sampling in the ADMIXTURE analysis.

Isolates Used in Sub-sampled ADMIXTURE analysis
ARG_3_5
ARG_4_2
AUS_FTA
CHE_07224
CHE_94202
CHN_11_133
CHN_17_18
CHN_1_62
CHN_21_2
CHN_45_6
CHN_5_93
CHN_6_69
CHN_9_2
CHN_E21
CHN_SD_5
FRA_B_STONE_95_45
GBR_JIW2
ISR_203
ISR_211
ISR_215
ISR_30p
ISR_66
ISR_7
ISR_K_U
JPN_244a
JPN_CHIKA
JPN_MEK_13b
KAZ_1a
POL_3
RUS_O1a
TUR_1c
USA_2
USA_7
USA_C4_6

Supplementary Table 10. Watterson's Theta and nucleotide diversity results for random eight isolates per population, using 10 kb windows in the whole genome.

	**Theta**	**Pi**
ARG	0.000282564	0.000272461
USA	0.000383662	0.000417275
JPN	0.000390569	0.000386048
CHNa	0.000418103	0.000439794
CHNh	0.000590866	0.000622678
CHN	0.000523810	0.000632756
EUR	0.000763978	0.000902355
ISR	0.000995946	0.001236648
ISRdic	0.000643996	0.000768240

**#ARG**	**#USA**	**#JPN**	**#CHNa**	**#CHNh**	**#CHN**	**#EUR**	**#ISR**	**#ISRdic**
ARG_3_10	USA_2	JPN_244a	CHN_13_76	CHN_12_3	CHN_10_8	CHE_07004	ISR_113	ISR_203
ARG_3_4	USA_3	JPN_244aii	CHN_15_9	CHN_12_50	CHN_15_17	CHE_10001	ISR_204	ISR_206
ARG_3_5	USA_5	JPN_CHIKA	CHN_17_40	CHN_18_11	CHN_1_62	CHE_94202	ISR_215	ISR_209
ARG_3_9	USA_7	JPN_CHI_79a	CHN_1_25	CHN_21_8	CHN_21_1	CHE_97266	ISR_218	ISR_211
ARG_4_1	USA_AK3_11	JPN_MEK_13a	CHN_21_2	CHN_35_1	CHN_21_2	FRA_SYROS_2000_15	ISR_30p	ISR_212
ARG_4_2	USA_C4_6	JPN_MEK_13c	CHN_51_3	CHN_36_3	CHN_49_1	GBR_JIW2	ISR_8	ISR_220
ARG_4_6	USA_J2_1	JPN_MEK_2b	CHN_E21	CHN_HB_21	CHN_7_8	POL_3	ISR_97	ISR_58
ARG_4_8	USA_Ken_2_5	JPN_CHI_79b	CHN_6_69	CHN_HB_22	CHN_SD_5	POL_6	ISR_K_U	ISR_66

Supplementary Table 11. RAiSD output statistics for cohort 172, without centromeres, using alleles with ancestral state known and keeping the data with a threshold above the 99.9%.

RaiSD (0.999)	count	min	max	average	median	sum kb	%of all chrom*	Genes found
ARG	2	1,628,174	1,676,314	1,652,244.00	1,652,244.00	3,304,488	2.40%	257
CHN	6	247,591	943,626	603,650.17	558,211.50	3,621,901	2.64%	170
CHNa	10	257,101	2,022,328	745,764.60	598,725.50	7,457,646	5.43%	536
EUR	5	168,108	774,224	392,940.40	359,200.00	1,964,702	1.43%	132
ISR	6	221,367	934,147	577,160.00	579,251.00	3,462,960	2.52%	137
ISRdic	40	209,648	1,527,046	498,331.35	448,764.50	19,933,254	14.51%	1219
JPN	4	635,373	1,287,633	908,765.75	856,028.50	3,635,063	2.65%	234
USA	4	857,210	1,223,272	1,096,820.25	1,153,399.50	4,387,281	3.19%	286
ALL (unique)	103	913	1,527,046	332,311.48	289,048.00	34,228,082	24.91%	2231

*excluding chromosome Bgt_chr-Un

Supplementary Notes

Supplementary Note 1. Discussion of the directionality of wheat powdery mildew from USA to Australia and vice versa.

Here, we discuss whether mildew migrated from Europe via the USA to Australia, or vice versa (via Australia to the USA). The genomic data itself does not imply directionality from USA to Australia, but it complements a strong historical case: if introduction had occurred from Europe through Australia to the USA, the ancient hybridization with a distant mildew strain would have had to occur in Australia, where wheat was introduced only in 1788[55], while it came to the USA already between 1600 and 1700. Furthermore, cultivation of *Hordeum* (little barley) is documented for native Americans[54]. Thus, a hybridization in Australia and a subsequent migration to the USA would require that the new hybrids completely out-competed earlier mildew strains in the USA. We, therefore, considered it more parsimonious that mildew hybridization occurred in the USA and that the descendants of these hybrids were then brought to Australia.

Supplementary Note 2. Gene flow is detectable between all wheat mildew populations

We used F4 statistics to assess whether there is gene flow between populations checking for allele sharing between all possible combinations of populations, designed as (pop1, pop2; pop3, *B.g. poae*/outgroup). We found significant F4 positive values, suggesting gene flow affecting all *B.g. tritici* populations, and even the different *forma specialis B.g. dicocci* (Supplementary Table 4).

Highest level of allele sharing (highest F4 value) was found between the JPN-CHNa populations. Conversely, we found only limited allele sharing (smaller F4 values) between the European and the other mildew populations, mostly with the populations that have recombined (e.g. the mildew population from China). The limited allele sharing could be due to a more recent separation of the mildew isolates from Europe, but it could also be due to the mapping reference being in the EUR population[31].

The Israeli *B.g. tritici* population showed a high number of singletons compared with other populations, despite indication for a bottleneck 50-100 generations ago. Since F4 indicated gene flow between *B.g. dicocci* and *B.g. tritici* (Supplementary Note 5), it is possible that the ISR *B.g. tritici* mildew population might still maintain more singletons compared to other populations with a recent bottleneck (e.g. the ARG population),

Supplementary Note 3. Details about the fastsimcoal2 scenarios.

In the three highest scoring fastsimcoal2 scenarios, the USA mildew population always separates from the EUR mildew population, and then there is substantial gene flow from the *B.g. dicocci* to the USA mildew population (Fig. 3c). These best scenarios have higher probabilities than other ones where the USA population derives only from the EUR population and does not have migration of the population (Supplementary Fig. 17 and Supplementary Table 5). All the different combinations of scenarios have the *B.g. dicocci* as an outgroup or placed next to the outgroup. The CHNa or the ISR populations seem to separate first from the *B.g. dicocci* in the Scenarios with the highest probability.

Supplementary Note 4. Details about the scans for signatures of selection results.

We observed that around a fourth of the genome is in the selection windows (Supplementary Table 11). ISRdic is the population with the most windows (40). The ISRdic population also has the most base pairs in selection windows.

Supplementary Note 5. F3 statistics could be helpful even if they are not conclusive.

We used admixture F3 statistic tests to check for recombination. The tests were of the type (pop1, pop2; JPN or CHNh). While it was negative and significant for the CHNh population case, proving recombination between CHNa and EUR population, it was positive and not significant for the JPN population. However, the closest results for combinations to the negative values were the ones with CHNa and USA as parents. This is suggesting that the East Asian ancestry found in China, along with the USA could be the most likely parents of the Japanese recombinant population (Supplementary Fig. 6). One reason why we did not obtain the expected negative F3 values, is that the exact parental strains are unknown. We only have their descendants, since mildew probably came from Asia approximately 2,000 years ago and has since been largely isolated in Japan.
