# Supplementary material for: Global genomic analyses of wheat powdery mildew reveal association of pathogen spread with historical human migration and trade
Source: Nat Commun. 2022 Jul 26;13:4315. doi: 10.1038/s41467-022-31975-0 (PMC9315327; doi:10.1038/s41467-022-31975-0)
Supplement: Supplementary file 2 — Description of Additional Supplementary Files [file 41467_2022_31975_MOESM2_ESM.pdf]

Title: Supplementary Data 1:
Description: Information about all the *Blumeria graminis tritici* and *dicocci* isolates used with geographical information, the sampling date, the host species they were found on and the number of the project the raw data can be found on. (Coordinates in italics are estimation of coordinates, while (p) in the Host Species refers to possibly, but not certainly being found on a *Triticum aestivum* host plant).

Title: Supplementary Data 2:
Description: Singletons results for random eight isolates per population. The specific isolates used are depicted on the left. The CHNa population is included in light green, while the CHNh is included in red font.

Title: Supplementary Data 3:
Description: All the fastsimcoal2 priors that were used for the various scenarios.
