# Supplementary material for: Global genomic analyses of wheat powdery mildew reveal association of pathogen spread with historical human migration and trade
Source: Nat Commun. 2022 Jul 26;13:4315. doi: 10.1038/s41467-022-31975-0 (PMC9315327; doi:10.1038/s41467-022-31975-0)
Supplement: Supplementary file 6 — Reporting Summary [file 41467_2022_31975_MOESM6_ESM.pdf]

nature portfolio

Corresponding author(s): Alexandros Georgios Sotiropoulos
Thomas Wicker

Last updated by author(s): Jul 7, 2022

Reporting Summary

Nature Portfolio wishes to improve the reproducibility of the work that we publish. This form provides structure for consistency and transparency in reporting. For further information on Nature Portfolio policies, see our Editorial Policies and the Editorial Policy Checklist.

Statistics

For all statistical analyses, confirm that the following items are present in the figure legend, table legend, main text, or Methods section.

n/a	Confirmed	
☐	☒	The exact sample size (*n*) for each experimental group/condition, given as a discrete number and unit of measurement
☐	☒	A statement on whether measurements were taken from distinct samples or whether the same sample was measured repeatedly
☐	☒	The statistical test(s) used AND whether they are one- or two-sided<br>*Only common tests should be described solely by name; describe more complex techniques in the Methods section.*
☐	☒	A description of all covariates tested
☐	☒	A description of any assumptions or corrections, such as tests of normality and adjustment for multiple comparisons
☐	☒	A full description of the statistical parameters including central tendency (e.g. means) or other basic estimates (e.g. regression coefficient) AND variation (e.g. standard deviation) or associated estimates of uncertainty (e.g. confidence intervals)
☐	☒	For null hypothesis testing, the test statistic (e.g. *F*, *t*, *r*) with confidence intervals, effect sizes, degrees of freedom and *P* value noted<br>*Give P values as exact values whenever suitable.*
☐	☒	For Bayesian analysis, information on the choice of priors and Markov chain Monte Carlo settings
☒	☐	For hierarchical and complex designs, identification of the appropriate level for tests and full reporting of outcomes
☐	☒	Estimates of effect sizes (e.g. Cohen's *d*, Pearson's *r*), indicating how they were calculated

Our web collection on statistics for biologists contains articles on many of the points above.

Software and code

Policy information about availability of computer code

Data collection	Downloaded data from ncbi (SRA). Does not apply.
Data analysis	There were various software and R packages used:<br>Homemade software: haplobloc software (https://github.com/wicker314/haplobloc), 4fdg analysis software (https://github.com/caldetas/vcf_4fold_degenerate_dating_haploid).<br>Published software: samtools (v1.9), vcftools (v0.1.5), msmc2 (v2.1.1), fastsimcoal2 (v2.6.0.3), plink (v1.9), trimmomatic (v0.38), bwa (v0.7.17-r1188), picard (v2.18.25-SNAPSHOT), qualimap (v.2.2.1), gatk (v.4.1.2.0), SPAdes (v3.12.0), ncbi+ blast (v2.7.1+), ADMIXTURE (v1.3), Treemix (v1.13), SnpEff/SnpSift (v4.3t), ADMIXTOOLS (v6.0), RAiSD (v2.9), pixy (v1.2.5.beta1), SplitsTree4 (v4.17.1).<br>R packages: adegenet (v2.1.3), memgene (v1.0.1), vcfR (v1.12.0), PopGenome (v2.7.5), StAMPP (v1.6.1), stats (v3.6.3), stats (v4.1.2), MASS (v7.3-53), ade4 (v1.7-15), vegan (v2.5-7), ggplot2 (v3.3.2).

For manuscripts utilizing custom algorithms or software that are central to the research but not yet described in published literature, software must be made available to editors and reviewers. We strongly encourage code deposition in a community repository (e.g. GitHub). See the Nature Portfolio guidelines for submitting code & software for further information.

Data

Policy information about availability of data

All manuscripts must include a data availability statement. This statement should provide the following information, where applicable:

- Accession codes, unique identifiers, or web links for publicly available datasets
- A description of any restrictions on data availability
- For clinical datasets or third party data, please ensure that the statement adheres to our policy

The whole-genome short read sequence data of the 87 newly sequenced mildew isolates generated in this study have been deposited in the NCBI's Short Read Archive (SRA) database under the accession code BioProject PRJNA625429 [https://www.ncbi.nlm.nih.gov/bioproject/PRJNA625429]. All mildew isolates' genomic sequences used on this study can be found on SRA ncbi and the accession codes can be found in the Supplementary Table 1 (PRJNA625429, SRP062198). The wheat powdery mildew isolates that have been sequenced here are available from the powdery mildew collection of Prof. Beat Keller (University of Zurich, bkeller@botinst.uzh.ch). Since individual mildew isolates may die over time, access to all isolates cannot be guaranteed indefinitely. Source data are provided with this paper.

Field-specific reporting

Please select the one below that is the best fit for your research. If you are not sure, read the appropriate sections before making your selection.

☐ Life sciences ☐ Behavioural & social sciences ☒ Ecological, evolutionary & environmental sciences

For a reference copy of the document with all sections, see nature.com/documents/nr-reporting-summary-flat.pdf

Ecological, evolutionary & environmental sciences study design

All studies must disclose on these points even when the disclosure is negative.

Study description	This is a population genetics study using various geographical populations of the grass mildew fungus with 173 isolates being finally used.
Research sample	The research sample is the grass powdery mildew fungus Blumeria graminis. Various formae speciales were used. The rationale was that we wanted to have a worldwide sample dataset. Extra data was used from SRA of ncbi to complete the genomic dataset. The dataset consists of whole-genome sequencing data.
Sampling strategy	Since it is quite hard to isolate mature and viable isolates of powdery mildew, isolates were chosen on the basis of availability. The sample size was sufficient for the planned analysis according to previous publications. Sample sizes were chosen on the basis of availability.
Data collection	To collect the data, co-authors of the paper went out into the field and collected a few wheat leaves infected with the mildew fungus or samples already collected before were added to the dataset from another mildew collection. The sample coordinates were recorded on using GPS tracking devices (e.g. cellphone) and were written with a pen on the envelopes that contained the biological samples.
Timing and spatial scale	Time wise data was mostly collected between 2010 and 2019, always during the end of the growing season of wheat, since mature chasmothecia that grow on dry wheat leaves were used for the sampling. A few samples were used from the 1990s. Sample collectors travelled to wheat fields around the world where wheat is growing and infected wheat powdery mildew. The spatial scale includes wheat fields from five continents (Europe, Asia, North America, South America, Australia).
Data exclusions	Data that was not of high quality was excluded (low sequencing coverage, clonal isolates). Isolates that were hard to propagate were also excluded from sequencing and further analyses.
Reproducibility	All analyses were repeated more than once with a slight change of mildew isolates and were fully reproducible.
Randomization	Isolates were either allocated into groups in geographical clusters or were grouped by admixture analyses output. Random mildew isolates and subgroups were used to repeat some of the analyses (where deemed important).
Blinding	Blinding was not applicable, since the goal of the study was to sample biodiversity of wheat powdery mildews.

Did the study involve field work? ☒ Yes ☐ No

Field work, collection and transport

Field conditions	Natural/Agricultural fields. Environmental factors (e.g. temperature, rain) were not relevant. Only the host plant (hexaploid and tetraploid wheat) were considered.
Location	All the different sampling locations are found in the Supplementary Table 1.
Access & import/export	Local collaborators and co-authors accessed the habitats, collected and shipped the samples to Switzerland. Where necessary we

	contacted the respective Nagoya Protocol authorities. For Argentina (Ministerio de Coordinación, GOBDIGI-104937111-621, 16-07-2020).
Disturbance	No disturbance was caused during the study, as the collection was done in a noninvasive way.

Reporting for specific materials, systems and methods

We require information from authors about some types of materials, experimental systems and methods used in many studies. Here, indicate whether each material, system or method listed is relevant to your study. If you are not sure if a list item applies to your research, read the appropriate section before selecting a response.

Materials & experimental systems

n/a	Involved in the study	
☒	☐	Antibodies
☒	☐	Eukaryotic cell lines
☒	☐	Palaeontology and archaeology
☐	☒	Animals and other organisms
☒	☐	Human research participants
☒	☐	Clinical data
☒	☐	Dual use research of concern

Methods

n/a	Involved in the study	
☒	☐	ChIP-seq
☒	☐	Flow cytometry
☒	☐	MRI-based neuroimaging

Animals and other organisms

Policy information about studies involving animals; ARRIVE guidelines recommended for reporting animal research

Laboratory animals	The study did not involve laboratory animals.
Wild animals	The study did not involve wild animals.
Field-collected samples	The powdery mildew fungus was collected from agricultural/natural fields.
Ethics oversight	The Nagoya Protocol on Access to Genetic Resources was used.

Note that full information on the approval of the study protocol must also be provided in the manuscript.
